# Molecular xenomonitoring of *Schistosoma mansoni* infections in *Biomphalaria choanomphala* at Lake Victoria, East Africa: Assessing roles of abiotic and biotic factors

**DOI:** 10.1371/journal.pntd.0012771

**Published:** 2025-01-02

**Authors:** Peter S. Andrus, Claire J. Standley, J. Russell Stothard, Christopher M. Wade

**Affiliations:** 1 School of Life Sciences, University of Nottingham, Nottingham, United Kingdom; 2 Center for Global Health Science and Security, Georgetown University, Washington, DC, United States of America; 3 Heidelberg Institute of Global Health, University of Heidelberg, Heidelberg, Germany; 4 Department of Tropical Disease Biology, Liverpool School of Tropical Medicine, Liverpool, United Kingdom; University of Oxford, UNITED KINGDOM OF GREAT BRITAIN AND NORTHERN IRELAND

## Abstract

Lake Victoria is a well-known hot spot for intestinal schistosomiasis, caused by infection with the trematode *Schistosoma mansoni*. The snail intermediate hosts of this parasite are *Biomphalaria* snails, with *Biomphalaria choanomphala* being the predominant intermediate host within Lake Victoria. The prevalence of *S. mansoni* infection within snail populations is influenced by both biotic and abiotic factors, including the physical and chemical characteristics of their environment, the incidence of infection in human populations (and reservoir hosts) and the level of genetic compatibility between the parasite and the host. Using molecular xenomonitoring, we measured the prevalence of *S. mansoni* infection within *B. choanomphala* populations along the Kenyan, Tanzanian and Ugandan shorelines of Lake Victoria and related this to the abiotic (habitat type, water depth, turbulence, temperature, conductivity, total dissolved solids, salinity, pH level) and biotic (*B. choanomphala* abundance, genetic diversity of host snail populations) factors of the lake. The overall mean prevalence of *S. mansoni* infection at Lake Victoria was 9.3%, with the highest prevalence of infection occurring on the Tanzanian shoreline (13.1%), followed by the Ugandan (8.2%) and Kenyan (4.7%) shorelines. There was a significant difference in *B. choanomphala abundance*, water temperature, conductivity, salinity, total dissolved solids and major anion/cation concentrations between the Kenyan, Tanzanian and Ugandan shorelines of Lake Victoria. A Spearman’s rank analysis found that the prevalence of *S. mansoni* infection had a significant, positive relationship with higher levels of *B. choanomphala* abundance, water acidity, and cation (Ca_2_^+^, Mg_2_^+^) concentrations. Additionally, we observed that sites with *S. mansoni* infection correlated with *B. choanomphala* populations with a higher mean haplotype diversity score compared to sites found without infection, though there was no significant relationship between the prevalence of infection and *B. choanomphala* haplotype diversity scores. Although our analysis is based upon an archival and unique collection of *Biomphalaria* snails, the abiotic and biotic relationships uncovered are useful for eco-epidemiological comparisons of intestinal schistosomiasis across Lake Victoria in future.

## Author summary

Human schistosomiasis is caused by the parasitic trematode genus, *Schistosoma*. The disease is transmitted when people come into contact with contaminated freshwater sources that contain snail intermediate host species carrying the parasite. The intestinal form of human schistosomiasis is commonly caused by *Schistosoma mansoni* which uses *Biomphalaria* snails as its intermediate host. To better understand the factors that affect the prevalence of *S. mansoni* infection within snail populations, the authors examined biotic and abiotic factors from sites across the Kenyan, Tanzanian and Ugandan shorelines of Lake Victoria. The study found that there was a significant relationship between high *S. mansoni* prevalence and several physiochemical factors such as water acidity and cation (Ca_2_^+^, Mg_2_^+^) levels. The study also observed that *B. choanomphala* populations with *S. mansoni* infection present had a higher number of private haplotypes and a higher mean haplotype diversity score when compared to populations without *S. mansoni* infection present. However, there was no significant relationship between the prevalence of *S. mansoni* infection and the genetic diversity of a population. Overall, this study improves our understanding of which factors influence *S. mansoni* infection among *Biomphalaria* populations.

## Introduction

Schistosomiasis is a parasitic disease caused by the intravascular parasite genus, *Schistosoma*. Schistosomiasis is a neglected tropical disease (NTD) that affects over 240 million people globally, with over 700 million people being at risk of infection [1]. The disease is endemic in 78 countries worldwide and seriously impacts developing countries, especially sub-Saharan Africa [2]. It is estimated that 3.3 million Disability-Adjusted Life Years (DALYs) were lost in 2010 due to urogenital or intestinal schistosomiasis [3]. The majority of intestinal schistosomiasis cases are caused by *Schistosoma mansoni* and its intermediate freshwater snail host, *Biomphalaria* [4,5]. East Africa is a well-known regional hotspot for schistosomiasis transmission with the disease being as prevalent as 2–18% in Kenya, 22–86% in Tanzania and 7–88% in Uganda [6]. The high prevalence of *S. mansoni* infection in East Africa is due to the large number of freshwater environments that *Biomphalaria* snails can inhabit, with the largest source of freshwater being Lake Victoria [7,8]. These favourable habitats combined with poor water hygiene and sanitation standards make the shoreline of Lake Victoria a hot spot for intestinal schistosomiasis [7]. *Biomphalaria* is notoriously invasive and are capable of rapidly expanding their territory due to their high fecundity and ability to self-fertilise [9]. This rapid expansion can lead to outbreaks of schistosomiasis as self-fertilisation and inbreeding leads to genetically homogeneous populations at the expense of schistosome resistance (as seen predominantly in *B. pfeifferi*) [10,11]. However, the distribution of *S. mansoni* is dependent on the ecological requirements of its intermediate host, with the availability of suitable freshwater habitats limiting the potential geographical reach of the parasite [8,12].

Two *Biomphalaria* species, *B. choanomphala* and *B. sudanica* have been reported to inhabit Lake Victoria and its surrounding shoreline [13–17]. Previous studies described these species as having either a “lacustrine” (*B. choanomphala*) or “non-lacustrine” (*B. sudanica*) morphology due to *B. choanomphala* being commonly found in deeper parts of the lake, and *B. sudanica* being commonly found in the swamps adjacent to the shoreline (S1 Fig) [7,18–22]. However, using molecular methods, both Standley et al. (2011) [23] and Zhang et al. (2018) [24] demonstrated that the *B. sudanica*-like snails in Lake Victoria were genetically more similar to *B. choanomphala* than to other *B. sudanica* populations found in Africa. This suggested that the *B. sudanica*-like snails from Lake Victoria were instead an ecophenotype (ecological phenotype) of *B. choanomphala*, with the morphological differences between the two snails being the result of individual populations adapting their shell morphology to fit their environment. This was expanded upon further by Andrus et al. (2023) [22] who found the *B. sudanica*-like snails in Lake Victoria were both genetically and morphologically separate from the *B. sudanica* snails found at Lake Albert. In this study, we follow Standley et al. (2011) [23], Zhang et al. (2018) [24] and Andrus et al. (2023) [22] and refer to these *B. sudanica*-like snails as *B. choanomphala*.

Webbe (1965) [25] and Prentice et al. (1970) [26] were the first to document that *B. choanomphala* snails at Lake Victoria were capable of transmitting *Schistosoma mansoni*. Subsequent parasitological surveys have consistently reported *B. choanomphala* snails (including *B. sudanica*-like snails from Lake Victoria) having a lower prevalence of *S. mansoni* infection (0.06–10%) when compared to other east African species, such as *B. pfeifferi* (0.2–10%), *B. stanleyi* (4.8–15%) and *B. sudanica* (0.2–13.3%) (excluding *B. sudanica*-like snails from Lake Victoria) [14–17,27–29]. *Schistosoma mansoni* infection can be determined using both traditional cercarial shedding methods [25] and molecular infection detection methods [30,31]. However, molecular detection methods are underutilised in detecting schistosome infection in intermediate snail hosts collected from the field [32]. *Biomphalaria* populations are known to be sensitive to a variety of abiotic factors in their habitat, which limits which environments they can inhabit [18,33]. Likewise, biotic factors such as the genetic diversity of a snail-host population can affect host susceptibility and parasite infectivity [34]. The ‘Red Queen’ hypothesis proposes that there is a continuous cycle of adaptation and counter-adaptation between populations of species that frequently interact with one another, such as parasites and hosts [35]. These reciprocal adaptive changes from host-parasite interactions can explain the genetic variability observed in host susceptibility and parasite infectivity [36]. Although the exact genetic mechanisms have not been fully identified in snail-schistosome systems, resistance and susceptibility in snails and infectivity of schistosomes have been shown to be heritable and maintained through cost-benefit trade-offs [37,38].

In this study, using molecular xenomonitoring, we investigate the prevalence of *S. mansoni* infection in *B. choanomphala* snails made from a set of five extensive expeditions sampling the Kenyan, Tanzanian and Ugandan shorelines of Lake Victoria. We examined the habitat type, water depth, turbulence, temperature, conductivity, total dissolved solids, salinity and pH level of the lake, as well as the abundance and genetic diversity of *B. choanomphala* snail populations. This was done to determine the effect abiotic and biotic factors have on the infection prevalence of *S. mansoni* in *B. choanomphala* populations across Lake Victoria. This study is the largest and only lake-wide collection of *B. choanomphala* snails from Lake Victoria that investigates the factors influencing the prevalence of *S. mansoni* infection.

## Materials and methods

### Collection sites

**Fig 1:**
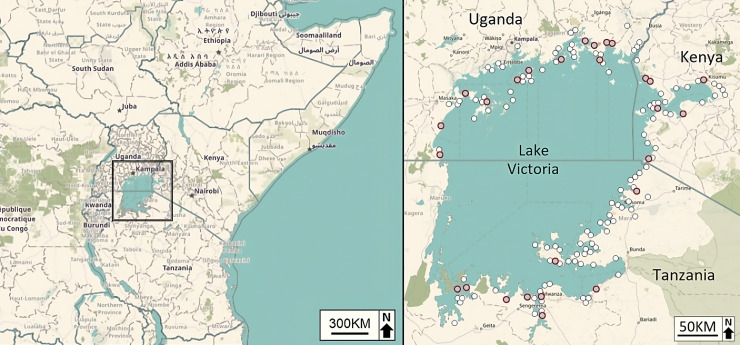
Map of East Africa (left) and the collection site locations around the shoreline of Lake Victoria (right). The 27 sites highlighted in grey were chosen to measure genetic diversity (this map was created using ‘OpenStreetMap’ https://www.openstreetmap.org).

Sampling was undertaken from 2008 to 2011 at 170 sites from the Kenyan (*n* = 35), Tanzanian (*n* = 82) and Ugandan (*n* = 53) shorelines of Lake Victoria (Fig 1), as part of malacological surveillance of the EU Framework 6 project entitled EU-CONTRAST [39]. Sampling sites were chosen opportunistically based upon accessibility, with common freshwater snail habitats such as marshes and the lake edge close to human settlements being chosen. Sampling was undertaken during the wet season, with samples collected during five field expeditions that took place over a two year period (Uganda: February to March 2008; Tanzania: June to July 2008; Tanzania and Kenya: January to February 2009; Uganda: January to February 2010; Uganda: August 2010). Sites were within 10 meters of the lakeshore and were approximately 10–20 km apart to ensure an even spread of sites along the Lake Victoria shoreline across Kenya, Tanzania and Uganda. The Lake Victoria shoreline is approximately 3,450 km (Kenya: 16%, Tanzania: 33%, Uganda: 51%) [40], and in total, we surveyed approximately 3,175 km of the shoreline. However, ~275 km of the western Tanzanian shoreline could not be surveyed due to inaccessibility.

At each site, the location was georeferenced using a handheld GPS device (Garmin GPS V, Garmin Ltd., Kansas City, USA) and the date, time and the weather conditions were documented. Next, qualitative measurements (habitat type, water depth, water turbulence, *B. choanomphala* abundance, and which shell morphotype were present) of the site were recorded *in situ*. The habitat type of a site was categorised as being either marshlands (type-a), lake edge (type-b) or other (type-c) (examples of snail habitats are shown in S2 Fig). If the site featured a combination of several habitat types, no more than three sub-categories were assigned. Water depth was assessed as being either shallow (<10 cm), moderately-shallow (10–30 cm), moderate (30–50 cm), moderately-deep (50–70 cm) and deep (>70 cm). Likewise, water turbulence was classified as being low (still, no movement), medium (some movement but limited wave action) or high (high disturbance, white caps were present). *Biomphalaria choanomphala* snails were sampled semi-quantitatively at each of the sites, with two collectors looking for snails for 15 minutes, using either a short or long-handled metal mesh scoop. *Biomphalaria choanomphala* abundance was measured as either being absent (zero snails), low (<10), medium (10–30) or high (>30). When found, *B. choanomphala* snails were collected and placed into jars filled with lake water for later processing. *Biomphalaria choanomphala* snails were identified using conchological identification methods as described by Mandahl-Barth (1962) [41] and Brown (1994) [18], collected snails had their shell morphologies recorded to see whether they exhibited non-lacustrine (morphotype-A) or lacustrine shell (morphotype-B) morphologies as described by Standley et al. (2011) [23] and Andrus et al. (2023a) [22] (S1 Fig). After processing, all snail samples were stored immediately in 100% ethanol for later DNA extraction. *Biomphalaria* collections were undertaken by Standley et al. (2012) [13] (field expeditions 1–4) and Rowel et al. (2015) [15] (field expedition 5), further information on these collections can be found in Standley et al. (2012) [13] and Rowel et al. (2015) [15].

Alongside the qualitative measurements, certain environmental factors such as water temperature (°C), conductivity (μS), total dissolved solids (g/L), salinity (g/L) and pH were measured at each site using a HI9813 handheld portable water meter (Hanna Instruments, Inc., Woonsocket, USA). These measurements were performed on site by taking the mean values from two separate measurements using two different water meters. Additionally, 15 ml of water was taken from each site, and was frozen for future detailed compositional analysis at the Natural History Museum, London. The concentrations of anions (fluoride, chloride, nitrate, phosphate and sulphate) in the water samples were determined using Reagent-Free Ion Chromatography (RFIC-EG), while the concentrations of cation (calcium, potassium, magnesium and sodium) were determined by Inductively Coupled Plasma Optical Emission Spectroscopy (ICP-OES). The equipment used to measure anion and cation concentrations were a ICS-3000 system (Dionex Inc., Sunnyvale, USA) and Varian Vista Pro axially viewed ICP-AES with a CCD detector and Varian SPS-5 autosampler (Varian, Inc., Palo Alto, USA), respectively. The water sample was tested twice and the mean value for each measurement was used. Further information about the abiotic data collection protocols can be found in Standley et al. (2012) [13].

### DNA extraction

Following collection, *B. choanomphala* snails were stored immediately in 100% ethanol. DNA was extracted using a modified CTAB (Cetyltrimethylammonium Bromide) extraction method (as described in Goodacre & Wade, 2001) [42]. Where numbers permitted, 12 *B. choanomphala* snails were extracted per site, while sites with fewer than 12 individuals had all of their snails extracted (for further information, please see S1 Table). After extraction, samples were resuspended in 100–200 µl of TE (10mM Tris-HCl, pH 8.0, 0.1mM EDTA) buffer and DNA yields were measured using a NanoPhotometer N50 (Implen, München, Germany). All DNA extracts were stored at −20 °C and were archived at the Liverpool School of Tropical Medicine until use.

### Molecular xenomonitoring of *S. mansoni* infection in *Biomphalaria choanomphala*

All of the extracted *B. choanomphala* samples were tested for *S. mansoni* infection between 2021 and 2022, using two different infection detection primer sets as described in Andrus et al. (2023b) [29]. The first primer set used was Sm^F/R^ designed by Sandoval et al. (2006) [30] (SM^F/R^-F: 5’-GAG ATC AAG TGT GAC AGT TTT GC-3’ and SM^F/R^-R: 5’-ACA GTG CGC GCG TCG TAA GC-3’). If the sample was positive, it was then tested using the ND5 primer set designed by Lu et al. (2016) [31] (ND5-F: 5’-ATT AGA GGC AAT GCG TGC TC-3’ and ND5-R: 5’-ATT GAA CCA ACC CCA AAT CA-3’) to determine whether the infection present in the snail was caused by *S. mansoni* or its closely-related sister species, *S. rodhaini*. The PCR reaction mixture and cycling conditions for the Sm^F/R^ and the ND5 primers were followed precisely as described by Sandoval et al. (2006) [30] and Lu et al. (2016) [31], respectively. The applicability of these methods for *S. mansoni* detection in *Biomphalaria* snails is discussed in Joof et al., 2020 [43]. Alongside the *B. choanomphala* samples, two negative controls (water and uninfected *B. glabrata* DNA) and two positive controls (pure *S. mansoni* DNA and infected *B. glabrata* DNA) were also included. These controls were provided by Professor Mike Doenhoff, School of Biology, University of Nottingham. Additionally, all DNA extracts were tested using the LSU-1iii/LSU-3iii primers [44] (LSU-1iii: 5’-TGC GAG AAT TAA TGT GAA TTG C-3’ and LSU-3iii: 5’-ACG GTA CTT GTC CGC TAT CG-3’) to ensure that the DNA was not degraded and was still amplifiable. The PCR cycling conditions for these primers were an initial denaturation at 96 °C for 2 min, followed by 35 cycles of 94 °C for 30 sec, 45 °C for 1 min, 72 °C for 2 min and a final extension step at 72 °C for 5 min. All PCR reactions were performed using Promega GoTaq G2 Master Mix. All PCR products were ran on a 2% agarose gel containing ethidium bromide and amplicons were observed under UV light. *Schistosoma mansoni* infection was confirmed based on whether a diagnostic band was present for both the Sm^F/R^ (~350 bp) and ND5 (~302 bp) primer sets.

### Population genetics of *Biomphalaria choanomphala*

Genomic DNA samples from 27 sites across Lake Victoria were selected to measure the genetic diversity and population structure of the *B. choanomphala* snails found across Lake Victoria. Selected sites were evenly distributed along the lakeshore and had a minimum of ten individuals. Population genetic analysis was done using 16S and COI genotyping, which used the 16Sarm/16Sbrm primer set [29] (16Sarm: 5’-CTT CTC GAC TGT TTA TCA AAA ACA-3’ and 16Sbrm: 5’-GCC GGT CTG AAC TCA GAT CAT-3’) and the universal COI primers designed by Folmer et al. (1994) [45] (LCO1490: 5’-GGT CAA CAA ATC ATA AAG ATA TTG G-3’ and HCO2198: 5’-TAA ACT TCA GGG TGA CCA AAA AAT CA-3’). All PCR reactions were performed using a 25 μl reaction volume containing 24 μl of PCR master mix (1U TAQ, 0.2 μM primers, 200 μM dNTP, 1.5 mM MgCl_2_) and 1 µl of DNA template. The PCR cycling conditions used for both the 16S and COI primer sets were identical, with an initial denaturation at 96 °C for 1minute, followed by 34 cycles of 94 °C for 1 min, 50 °C for 1 min, 72 °C for 1 min and a final extension at 72 °C for 10 mins. PCR products were ran on a 2% agarose gel containing ethidium bromide and observed under UV light, with PCR products purified and sequenced by either the Natural History Museum, London or using Macrogen’s EZ Seq service.

### Bioinformatics and statistical analysis

*Biomphalaria choanomphala* sequences were aligned using the MUSCLE (Multiple Sequence Comparison by Log-Expectation) algorithm in the program Seaview v5 [46], with misaligned sections of the 16S and the COI fixed by hand and sites for tree building selected using the Gblocks program [47]. Phylogenetic trees were constructed using the Maximum Likelihood method, using a General Time Reversible model incorporating gamma correction (GTR+Γ) in the program MEGA v11 [48], with bootstrap analysis undertaken using 1000 replicates. DNASP v6 [49] was used to determine haplotype (gene) diversity scores (Hd), nucleotide diversity (π) and to examine population structure among populations between countries using Wright’s F-statistics (F_st_). Pairwise distances were calculated with MEGA 11 using the Maximum Composite Likelihood method [48].

Due to the non-normal distribution of our dataset, several non-parametric tests were performed using SPSS v26 (IBM, Armonk, USA) [50]. A two-tailed bivariate Spearman’s rank correlation analysis was performed to determine the relationships between *B. choanomphala* abundance, snail host haplotype diversity, prevalence of *S. mansoni* infection, and the abiotic factors of Lake Victoria. Similarly, a Kruskal–Wallis test (followed by a post-hoc Dunn’s test), a Mann–Whitney U test, and a Pearson’s chi-squared (X^2^) test (with Yates’ correction) were performed to compare the abundance of *B. choanomphala*, haplotype diversity, prevalence of infection and abiotic factors between the Kenyan, Tanzanian and Ugandan shorelines of Lake Victoria.

### GenBank accessions

All of the 16S and COI sequences of *B. choanomphala* used in this study are available on GenBank. The *B. choanomphala* 16S gene sequences are available in accession numbers HM768950-HM769131 and OQ924869-OQ924928. Likewise, the *B. choanomphala* COI gene sequences are available in accession numbers HM769132-HM769258 and OQ849937-OQ849996. For further information, see S2 Table.

## Results

### *Biomphalaria choanomphala* Abundance at Lake Victoria

**Fig 2:**
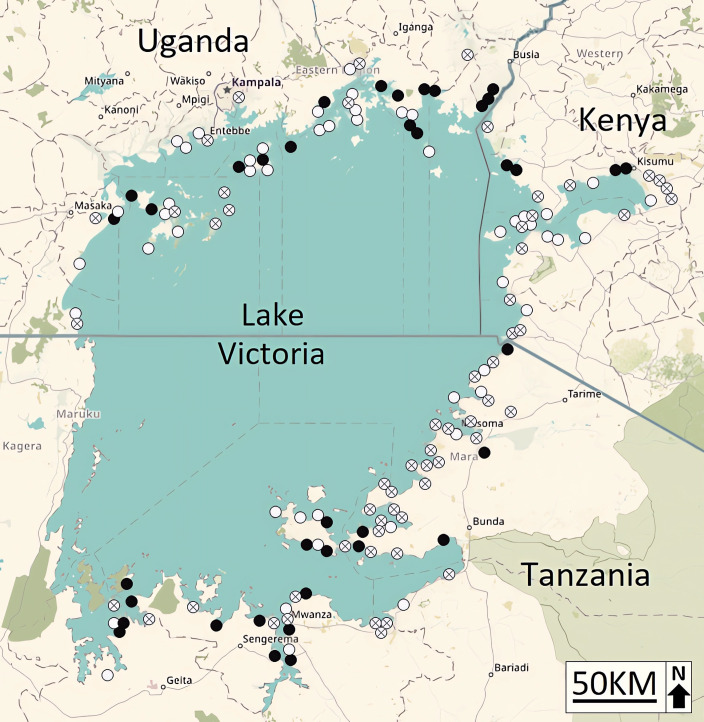
Map of collection sites at Lake Victoria showing where *Biomphalaria choanomphala* snails were found, and the incidence of *Schistosoma mansoni* infection in these snail populations. Collection sites with no *B. choanomphala* are shown with a cross. White circles denote sites where *B. choanomphala* snails were found, but were uninfected with *S. mansoni*. Black circles denote sites where *S. mansoni* infected *B. choanomphala* snails were found (created using ‘OpenStreetMap’ https://www.openstreetmap.org).

*Biomphalaria choanomphala* snails were present at 107 of the 170 sites surveyed at Lake Victoria (Fig 2 and S1 Table). Of these 107 sites, 44 had a low abundance (<10) of *B. choanomphala*, 25 had a medium abundance (10–30), and 38 had a high abundance (>30; Table 1). The Ugandan sites had the highest abundance of *B. choanomphala* snails, followed by the Tanzanian and Kenyan sites (Table 1). When categorised by morphotype, we found 52 sites had morphotype-A and 44 sites had morphotype-B (Table 1). Only 11 sites had both morphotypes present, with the majority of these sites being lake-marsh ecosystems on the Ugandan and the Tanzanian shorelines (Table 1). We found morphotype-A snails were more prevalent than morphotype-B snails at the Kenyan and Tanzanian sites, while morphotype-B snails were more prevalent at the Ugandan sites (Table 1). When categorised by habitat type, *B. choanomphala* snails were present at 40 marshland sites (a), 50 lake edge sites (b) and the remaining 17 sites were a mixture of different ecosystems (c) such as canals, rice paddies, ponds bordering the lake, and other hybrid environments (Table 1 and S1 Table and S2 Fig).

**Table 1:** Summary of the *B. choanomphala* abundance and abiotic factors collected across the Kenyan (*n* = 35), Tanzanian (*n* = 82) and Ugandan (*n* = 53) sites of Lake Victoria.

	Category	Number of sites		
		Kenya	Tanzania	Uganda
*B. choanomphala* Abundance	No Snails	14	37	12
	Low (<10)	9	24	11
	Medium (11–30)	9	4	12
	High (>30)	3	17	18
Ecophenotypes	No Snails	14	37	12
	Only Morphotype-A	19	30	3
	Only Morphotype-B	2	13	29
	Both Morphotypes	0	2	9
Habitat	Marsh (a)	12	30	9
	Lake Edge (b)	14	35	36
	^*^Other (c)	9	17	8
Water Depth	Shallow (<10 cm)	15	48	22
	Mod-Shallow (10–30 cm)	3	4	8
	Moderate (30–50 cm)	7	12	9
	Mod-Deep (50–70 cm)	2	2	7
	Deep (>70 cm)	7	8	5
	Missing (N/A)	1	8	2
Water Turbulence	Low	28	47	12
	Medium	2	7	9
	High	4	9	9
	Missing (N/A)	1	19	23

Note: Water turbulence: Low = still, no movement, Medium = some movement, but limited wave action, and High = high disturbance, white caps were present.

* Breakdown of other (c) habitats are as follows: Ditch-Marsh: 1, Ditch-Pond: 2, Ditch-Rice paddy: 1, Lake-Marsh: 15, Lake-Stream: 1, Marsh-Field: 3, Pond: 8, Pond-Rice paddy: 1, and Rice paddy: 2.

### Prevalence of *S. mansoni* infection at Lake Victoria

Of the 107 sites with *B. choanomphala* present, we found *S. mansoni* infection at 35.5% of sites (38 of 107 sites) (Fig 2). All of our Sm^F/R^ positive *Biomphalaria* samples were confirmed to be infected with *S. mansoni* as every sample gave a diagnostic band length of ~302 bp when tested with the ND5 primer set. The Tanzanian shoreline had the highest number of infected sites, with 40% of the sites with *B. choanomphala* snails present (18/45) infected with *S. mansoni*. This was followed closely by the Ugandan shoreline with 39% (16/41) of sites with *B. choanomphala* infected and the Kenyan shoreline with only 19% (4/21) of sites with *B. choanomphala* infected (Fig 2 and S1 Table). A Kruskal–Wallis test found there was no significant difference in the number of infected sites across the Kenyan, Tanzanian and Ugandan shorelines, X^2^ ([2], *n* = 107) = 3.07, *p* = .215. The sites with the highest number of infected *B. choanomphala* snails were T027b (7 of 10 snails) and T033a (4 of 10 snails), with the remaining sites having a maximum of two (or less) infected snails (S1 Table). Of the 40 marshland sites with *B. choanomphala* snails (a), 14 had infection present (35%), while 16 of the 50 lake edge sites with *B. choanomphala* snails (b) had infection present (32%) and the mixed sites (c) had infection present at 8 of the 17 sites (47%) with *B. choanomphala* snails. A Kruskal–Wallis test found there was no significant difference in the number of infected sites at each of the three ecosystems (X^2^ [[2], *n* = 635] = 1.25, *p* = .535).

When partitioned by number of infected snails, the overall prevalence of *S. mansoni* infection for Lake Victoria was 9.3%, with 59 of the 635 *B. choanomphala* snails testing positive for *S. mansoni* infection. The Tanzanian shoreline of Lake Victoria had the highest mean prevalence of infection with 13.1% (31/237) of snails infected, followed by the Ugandan shoreline with 8.2% (22/269) and the Kenyan shoreline with 4.7% (6/129). Likewise, a Kruskal–Wallis test indicated that there was no significant difference in the number of infected snails found at the Kenyan, Tanzanian and Ugandan shorelines, X^2^ ([2], *n* = 635) = 4.05, *p* = .132. Lastly, when categorised by morphotype, the morphotype-A form of *B. choanomphala* had an infection prevalence of 7.8% (27/347), while the morphotype-B form had an infection prevalence of 10.8% (32/288). A chi-square test of independence was performed to examine the relation between shell morphotype and infection. There was no significant association between the two *B. choanomphala* morphotypes and the prevalence of *S. mansoni* infection (X^2^ (1, *n* = 635) = 1.69, *p* = 1.93).

### Host snail genetic diversity and its effect on infection prevalence

Of the 27 sites selected for host snail population genetic analysis, 166 unique 16S haplotypes (*n* = 306) and 114 unique COI haplotypes (*n* = 313) were found (S3 Fig). The mean haplotype diversity (Hd) scores of the 27 sites were 0.845 (± 0.16) for 16S, and 0.787 (± 0.17) for COI. The mean nucleotide diversity (π) value for all Lake Victoria sites was 0.015 (± 0.009) for 16S, and 0.008 (± 0.005) for COI. The overall range of pairwise distances of the 27 sites genotyped at Lake Victoria was 0.0–3.5% for the 16S and 0.0–4% for the COI. When categorised by morphotype, we found 12 of the 16S (H9, H17, H48, H51, H60, H65, H69, H79, H110, H111, H160, H183) and 12 of the COI (H1, H5, H7, H8, H23, H27, H32, H42, H46, H48, H61, H64) haplotypes commonly found throughout Lake Victoria exhibited both morphotype-A and morphotype-B morphologies (S2 Table).

When partitioned by shoreline, we found the Ugandan shoreline had the highest number of haplotypes with 99 16S haplotypes (*n* = 151) and 60 COI haplotypes (*n* = 156). Of the 12 Ugandan sites sampled, the mean Hd score was 0.883 (±0.19) for 16S and 0.747 (±0.19) for COI. The mean nucleotide diversity value for Ugandan sites was 0.018 (±0.01) for 16S, and 0.007 (±0.005) for COI. Pairwise distances for the Ugandan sites were 0.0–3.1% for 16S and 0.0–3.9% for COI. The Tanzanian shoreline had the second highest number of haplotypes with 50 16S haplotypes (*n* = 93) and 38 COI haplotypes (*n* = 93). Of the nine Tanzanian sites sampled, the 16S and COI had a mean Hd score of 0.863 (±0.09) and 0.802 (±0.16), respectively. The mean nucleotide diversity value for Tanzanian sites was 0.016 (±0.008) for 16S, and 0.008 (±0.005) for COI. Pairwise distances for the Tanzanian sites were 0.0–3.4% for 16S and 0.0–3.8% for COI. Lastly, the Kenyan shoreline had the lowest number of haplotypes with 24 16S haplotypes (*n* = 62) and 24 COI haplotypes (*n* = 64). Of the six Kenyan sites sampled, the mean Hd score was 0.748 (± 0.16) for 16S and 0.844 (± 0.07) for COI. The mean nucleotide diversity value for Kenyan sites was 0.007 (± 0.003) for 16S, and 0.009 (± 0.006) for COI. Pairwise distances for the Kenyan sites were 0.0–1.9% for 16S and 0.0–1.2% for COI.

When comparing the amount of haplotype diversity (Hd) at the 13 sites found with infection against the 14 sites found without infection, we found sites with infection had a higher mean Hd score than sites without infection (S3 Table). The mean Hd score of the 13 *B. choanomphala* collection sites with infection was 0.881 (± 0.1) for 16S and 0.841 (± 0.11) for COI, while the mean Hd score of the 14 collection sites with no infection was 0.814 (± 0.2) for 16S and 0.737 (± 0.19) for COI (Table 2). However, a Mann–Whitney U test found this difference in mean Hd score was not significant for either the 16S (U = 93.5, *p* = 0.903) or the COI (U = 118.5, *p* = 0.182). When partitioned by country, we find not all of the countries share this trend of sites with infection having a higher mean Hd score than sites without infection. For example, the mean Hd score of the 16S was higher for sites found without infection (0.933) than sites found with infection (0.828) on the Tanzanian shoreline (Table 2). Likewise, the mean Hd score of the COI was higher for sites found without infection (0.879) than sites found with infection (0.774) on the Kenyan shorelines (Table 2). A Spearman’s rank correlation test found that there was positive correlation between haplotype diversity scores and the prevalence of *S. mansoni* infection for both the 16S (*R*_*s*_ = 0.003) and COI (*R*_*s*_ = 0.229). However, the correlations between haplotype diversity scores and the prevalence of *S. mansoni* infection were not statistically significant for both the 16S (*p* = 0.989) and COI (*p* = 0.251).

**Table 2:** Comparing the mean haplotype diversity (Hd) scores of Lake Victoria sites found with and without *S. mansoni* infection.

	Mean Haplotype Diversity Scores (± SD)			
	16S		COI	
	Uninfected	Infected	Uninfected	Infected
Overall mean (*n* = 27)	0.814 (+ 0.2)	0.881 (± 0.1)	0.737 (+ 0.2)	0.841 (± 0.1)
Kenya (*n* = 6)	0.691 (± 0.1)	0.862 (± 0.1)	0.879 (± 0.0)	0.774 (± 0.1)
Tanzania (*n* = 9)	0.933 (± 0.0)	0.828 (± 0.1)	0.715 (± 0.2)	0.846 (± 0.1)
Uganda (*n* = 9)	0.833 (± 0.0)	0.953 (± 0.0)	0.666 (± 0.2)	0.862 (± 0.1)

When measuring the population structure (F_st_) between *B. choanomphala* populations using the 16S, we found the population structure was highest among the Kenyan and Ugandan populations (0.305), followed by the Tanzanian and Ugandan populations (0.242), while the Kenyan and Tanzanian populations had the lowest amount of structure (0.098) (Table 3). Likewise for the COI, we found the population structure was highest among the Kenyan and Ugandan populations (0.195). However, the second highest F_st_ value was between the Kenyan and Tanzanian populations (0.118), followed by the Tanzanian and Ugandan populations (0.067) (Table 3). Next, we measured the population structure between *B. choanomphala* populations found with *S. mansoni* infection between countries. We found the F_st_ values were highest between Kenyan and Ugandan sites for the 16S (0.490), followed by the Tanzanian and Ugandan sites (0.369), and lastly, the Kenyan and Tanzanian sites (0.192) had the lowest values (Table 3). However, when using the COI, we found the Kenyan and Tanzanian sites (0.210) had the highest values, followed by the Kenyan and Ugandan sites (0.123), and lastly, the Tanzanian and Ugandan sites (0.104) had the lowest value (Table 3). Likewise, when we measured the population structure between *B. choanomphala* populations found without *S. mansoni* infection between countries, we found the F_st_ values were the highest between Kenyan and Ugandan sites (16S: 0.474; COI: 0.452), followed by the Tanzanian and Ugandan sites (16S: 0.340; COI: 0.287) and the Kenyan and Tanzanian sites (16S: 0.137; COI: 0.152) (Table 3).

**Table 3:** Comparing the F_st_ values of *B. choanomphala* populations across Lake Victoria.

Population 1	Population 2	F_st_ Value	
		16S	COI
Kenya	Tanzania	0.098	0.118
Kenya	Uganda	0.305	0.195
Tanzania	Uganda	0.242	0.067
Infected Kenya	Infected Tanzania	0.192	0.210
Infected Kenya	Infected Uganda	0.490	0.123
Infected Tanzania	Infected Uganda	0.369	0.104
Uninfected Kenya	Uninfected Tanzania	0.137	0.152
Uninfected Kenya	Uninfected Uganda	0.474	0.452
Uninfected Tanzania	Uninfected Uganda	0.340	0.287

**Fig 3:**
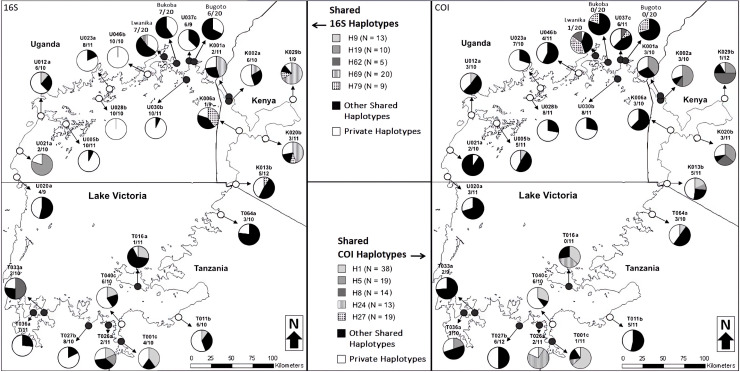
Distribution of private and shared 16S and COI haplotypes of *B. choanomphala* populations (*n* = 27) found at Lake Victoria. Private haplotypes are coloured white in the pie chart, while shared haplotypes are shaded. Sites found without *S. mansoni* infection are shown with a white dot (*n* = 14), while sites found with infection are shown with a black dot (*n* = 13). This map was hand traced using the ‘Standard map’ from ‘OpenStreetMap’ (https://www.openstreetmap.org) as the base layer.

Lastly, we mapped the distribution of private and shared 16S and COI haplotypes of *B. choanomphala* throughout Lake Victoria (Fig 3). The mean percentage of private haplotypes found within each *B. choanomphala* population was 46.7% for 16S haplotypes and 29.5% for COI haplotypes, while the mean percentage of shared haplotypes was higher for both the 16S (53.3%) and COI (70.5%). We found the Kenyan sites had the highest mean percentage of shared haplotypes with 71% for the 16S and 71.9% for the COI. Next, were the Tanzanian sites which had the second highest mean percentage of shared haplotypes with 58% for the 16S and 70.5% for the COI. The Ugandan sites had the lowest mean percentage of shared haplotypes with 43% for the 16S and 69.9% for the COI. When comparing the rate of shared haplotypes between *B. choanomphala* populations found with and without *S. mansoni* infection, we found sites with infection had more shared haplotypes for both the 16S (58.6%) and COI (78.9%) than sites found without infection (16S: 47.2%; COI: 61.1%) (Fig 3). However, we found on average the uninfected Kenyan sites had more shared haplotypes for both the 16S (75.6%) and COI (70%) than infected sites (16S: 61.9%; COI: 72.7%). Conversely, on average the infected Tanzanian sites had more shared haplotypes for both the 16S (61.9%) and COI (78.2%) than uninfected sites (16S: 50%; COI: 54.8%). Likewise, on average the infected Ugandan sites had more shared haplotypes for both the 16S (55%) and COI (81.7%) than uninfected sites (16S: 29.6%; COI: 56.8%).

### Analysis of infection prevalence, *B. choanomphala* abundance and abiotic factors

As mentioned previously, there was no significant difference in the prevalence of infection between the three shorelines of Lake Victoria. However, when testing the abundance of *B. choanomphala* snails between the Kenyan, Tanzanian and Ugandan shorelines, a Kruskal–Wallis found there was a significant difference between the three shorelines, X^2^ ([2], *n* = 170) = 11.41, *p* = .003. Similarly, there was a significant difference in the abundance of both morphotype-A (X^2^ [[2], *n* = 170] = 6.62, *p* = .036) and morphotype-B (X^2^ [[2] *n* = 170] = 59.13, *p <* .001) *B. choanomphala* snails across the three shorelines. When testing the abiotic factors between the Kenyan, Tanzanian and Ugandan shorelines, it found that the water temperature (X^2^ [(2) *n* = 165] = 18.07, *p* < .001), conductivity (X^2^ [(2) *n =* 166] = 42.19, *p* < .001), total dissolved solids (X^2^ [[2] *n =* 164] = 42.16, *p* < .001), salinity (X^2^ [[2] *n* = 154] = 26.87, *p* < .001), fluoride (X^2^ [[2] *n* = 141] = 57.99, *p* < .001), phosphate (X^2^ [[2] *n* = 141] = 8.27, *p* = .016), sulphate (X^2^ [[2] *n* = 141] = 39.12, *p* < .001), sodium (X^2^ [[2] *n* = 140] = 23.58, *p* < .001) and potassium (X^2^ [[2] *n* = 139] = 6.75, *p* = .034) levels were significantly different at each of the three shorelines. Conversely, the remaining abiotic factors, such as water pH (*p* = .354), chloride (*p* = .486), nitrate (*p* = .067), magnesium (*p* = .879) and calcium (*p* = .461) levels showed no significant difference between the three shorelines of Lake Victoria. The Dunn’s post-hoc test (using a Bonferroni correction) output of each Kruskal–Wallis test performed can be found in Table 4.

**Table 4:** The Kruskal–Wallis Dunn’s post-hoc test output of the infection prevalence, *B. choanomphala* abundance and the abiotic factors of the Kenyan (*n* = 35), Tanzanian (*n* = 82) and Ugandan (*n* = 53) shoreline collection sites.

	Kenya–Tanzania		Kenya–Uganda		Tanzania–Uganda	
	Mean Rank	*p* value	Mean Rank	*p* value	Mean Rank	*p* value
Infection	−8.66	0.23	−15.64	0.44	−6.97	0.27
*B. choanomphala* Abund.	1.90	0.84	−24.98	0.01	−26.89	0.00
Morphotype-A Abund.	11.68	0.17	23.54	0.01	11.86	0.11
Morphotype-B Abund.	−9.92	0.23	−58.51	<.001	−48.58	<.001
Temperature (°C)	36.48	<.001	9.11	0.39	−27.38	<.001
Conductivity (μS)	17.89	0.06	62.71	<.001	44.81	<.001
pH	11.85	0.23	2.06	0.85	−9.79	0.25
Total Dissolved Solids (g/L)	12.44	0.21	59.66	<.001	47.22	<.001
Salinity (g/L)	4.77	0.51	32.79	<.001	28.02	<.001
Fluoride (F^−^)	22.58	<.001	69.77	<.001	47.19	<.001
Chloride (Cl^−^)	8.55	0.32	10.99	0.25	2.44	0.76
Nitrate (NO_3_^−^)	13.38	0.27	16.70	0.13	3.32	0.75
Phosphate (PO_4_^3−^)	7.19	0.39	25.16	<.001	17.96	0.02
Sulphate (SO_4_^3−^)	29.01	<.001	59.84	<.001	30.83	<.001
Sodium (Na^+^)	18.41	0.03	45.43	<.001	27.01	<.001
Magnesium (Mg_2_^+^)	4.28	0.61	3.69	0.69	−0.59	0.94
Calcium (Ca_2_^+^)	3.37	0.70	11.22	0.24	7.85	0.34
Potassium (K^+^)	10.58	0.22	−10.13	0.29	−20.71	0.01

Note: Non-significant differences were greyed out. The *p* values displayed are adjusted using Bonferroni corrections.

When comparing the abundance of *B. choanomphala* snails between the three shorelines, we found the Ugandan shoreline had a significantly higher abundance of *B. choanomphala* snails when compared to the Kenyan and Tanzanian sites (Table 4). When comparing the abundance of each morphotype, we found the abundance of morphotype-A snails was significantly higher only at the Kenyan shoreline when compared to the Ugandan shoreline (Table 4). However, the abundance of morphotype-B snails was significantly higher at Ugandan shorelines when compared to both the Kenyan and Tanzanian shorelines (Table 4).

When comparing the abiotic factors collected at each shoreline, we found the Kenyan and Tanzanian shorelines were the most similar. The only significant differences between the Kenyan and Tanzanian shorelines were that the Kenyan sites had a higher median water temperature, fluoride, sulphate, and sodium levels than the Tanzanian sites (Table 4 and S4 Table). When comparing the Kenyan and Ugandan shorelines, the median water conductivity, TDS, salinity, fluoride, phosphate, sulphate and sodium levels were significantly higher at the Kenyan sites than the Ugandan sites (Table 4). Lastly, when comparing the Tanzanian and Ugandan shorelines, the median conductivity, TDS, salinity, fluoride, phosphate, sulphate and sodium levels were significantly higher at the Tanzanian sites than at the Ugandan sites (Table 4). However, the median water temperature and potassium levels were significantly higher at the Ugandan sites than the Tanzanian sites (Table 4).

A Spearman’s rank correlation analysis found *B. choanomphala* abundance had several significant relationships with chloride (0.354), magnesium (0.322), phosphate (0.319), potassium (0.316), pH (−0.311), calcium (0.238), nitrate (0.215) and water turbulence (−0.214) (Table 5). Likewise, morphotype-A abundance had a significant negative relationship with morphotype-B abundance (−0.177) and *vice versa*. (Table 5). This relationship indicates that each morphotype prefers inverse environmental factors to one another. For example, morphotype-A abundance had a significant positive relationship with sulphate (0.508), water conductivity (0.421), nitrate (0.404), sodium (0.402), calcium (0.398), phosphate (0.394), chloride (0.379), TDS (0.336), magnesium (0.307), salinity (0.252) and potassium (0.241). Whereas morphotype-B abundance has a significant negative relationship with sulphate (−0.359), water conductivity (−0.363), nitrate (−0.181), sodium (−0.316), TDS (−0.379), salinity (−0.256) and fluoride (−0.391) (Table 5). Moreover, morphotype-A abundance had a significant negative relationship with water turbulence (−0.447), pH (−0.386) and water depth (−0.170), while morphotype-B abundance had a significant positive relationship with water turbulence (0.269) and water depth (0.161) (Table 5). Lastly, there were several significant relationships with prevalence of infection, such as *B. choanomphala* abundance (0.445), morphotype-B abundance (0.306), morphotype-A abundance (0.271), pH (−0.199), calcium (0.184) and magnesium (0.175) concentrations (Table 5).

**Table 5:** Spearman’s rank correlation coefficients relating prevalence of *S. mansoni*, *B. choanomphala* abundance and abiotic factors of the Kenyan (*n* = 35), Tanzanian (*n* = 82) and Ugandan (*n* = 53) shoreline collection sites.

	Sites (*n=*)	Correlation coefficient			
		Infection	Abundance	Morphotype-A	Morphotype-B
Infection	170	–	–	–	–
*B. choanomphala* Abund.	170	0.445**	–	–	–
Morphotype-A Abund.	170	0.271**	0.619**	–	–
Morphotype-B Abund.	170	0.306**	0.563**	−0.177*	–
Conductivity	166	0.064	0.125	0.421**	−0.363**
pH	165	−0.199*	−0.311**	−0.386**	−0.032
Temperature	165	0.075	0.067	0.063	0.041
TDS	164	−0.023	0.050	0.336**	−0.379**
Water Depth	159	0.009	−0.058	−0.170*	0.161*
Salinity	154	0.060	0.064	0.252**	−0.256**
Fluoride (F^-^)	141	−0.092	−0.144	0.154	−0.391**
Chloride (Cl^−^)	141	0.154	0.354**	0.379**	0.013
Phosphate (PO_4_^3−^)	141	0.113	0.319**	0.394**	−0.064
Sulphate (SO_4_^3−^)	141	−0.004	0.157	0.508**	−0.359**
Sodium (Na^+^)	140	0.131	0.139	0.402**	−0.316**
Magnesium (Mg_2_^+^)	140	0.175*	0.322**	0.307**	0.009
Calcium (Ca_2_^+^)	140	0.184*	0.238**	0.398**	−0.155
Potassium (K^+^)	139	0.159	0.316**	0.241**	0.140
Nitrate (NO_3_^−^)	134	−0.041	0.215*	0.404**	−0.181*
Water Turbulence	127	−0.105	−0.214*	−0.447**	0.268**

Note: Non-significant correlations were greyed out; * indicates a significance of *p* < 0.05 and ** indicates a significance of *p* < 0.001.

## Discussion

Our molecular xenomonitoring study with multivariate analyses of available abiotic and biotic data investigated the prevalence of *S. mansoni* infection in *B. choanomphala* snails across Lake Victoria. Specifically, we addressed whether genetic diversity and abiotic (temperature, pH, physiochemical parameters etc.) factors had an effect on schistosome infection prevalence. As previously mentioned in the introduction, we collectively refer to the *B. choanomphala* and *B. sudanica*-like snails at Lake Victoria as a single species that express two distinct ecophenotypes [22–24]. Standley et al. (2014) [51] reported that *B. choanomphala* snails found at Lake Victoria had high levels of genetic diversity, high levels of both inter- and intra-population diversity, low levels of gene flow between populations and low levels of inbreeding. They theorised that this high level of genetic diversity could be caused by several factors relating to the environment (homogeneous habitats), human activity (mass treatment and snail control programs) and *S. mansoni* infection. However, Standley et al. (2014) [51] were unable to examine whether *S. mansoni* infection prevalence was influenced by *B. choanomphala* population structure due to the lack of data on whether a snail was infected or not. Our study provides this missing infection data and incorporates it with snail host genetic diversity and abundance data as well as data on abiotic characteristics. Our study is currently the only survey of *S. mansoni* infection in *B. choanomphala* snails that encompasses all three shorelines of Lake Victoria.

### Prevalence of *S. mansoni* infection in *B. choanomphala* snails in Lake Victoria

Our study found a mean prevalence of *S. mansoni* infection of 9.3% in *B. choanomphala* snails at Lake Victoria, with the highest prevalence of infection observed on the Tanzanian shoreline, followed by the Ugandan and Kenyan shoreline, respectively. Our study found a higher mean prevalence of *S. mansoni* infection when compared to previous parasitological studies. Previously, Gouvras et al. (2017) [16] reported 1.2% of snails on the Tanzanian shoreline were shedding cercariae, while 1.8–2.1% were shedding on the Ugandan shoreline [15,27] and 0.7–1.5% were shedding on the Kenyan shoreline [52,53]. The reason for this increase in infection prevalence is most likely attributed to the use of molecular methods to detect infection in the present study rather than the traditional cercarial shedding method. Molecular detection methods tend to show a higher number of infected snails as they are able to detect infection in both prepatent and actively shedding snails and are thus less likely to give false negative results [31,43,54].

When categorised by morphotype, we found the morphotype-B form of *B. choanomphala* had a higher mean infection prevalence than the morphotype-A form. Similarly, we found morphotype-B variants of *B. choanomphala* had a stronger relationship with *S. mansoni* infection than morphotype-A snails. However, this difference in infection prevalence was not statistically significant. Consistent with our findings, Mutuku et al. (2021) [17] reported that *S. mansoni* infection and cercarial production was significantly higher in *B. choanomphala* (morphotype-B) snails than the *B. sudanica*-like (morphotype-A) snails found at Lake Victoria, regardless of miracidium dosage or whether the eggs came from allopatric or sympatric sources. However, Rowel et al. (2015) [15] and Gouvras et al. (2017) [16] found the opposite trend, with the *B. sudanica*-like (morphotype-A) snails at Lake Victoria having a higher *S. mansoni* infection prevalence than the *B. choanomphala* (morphotype-B) snails.

### The effect abiotic factors have on *B. choanomphala* abundance and shell morphology

A Spearman’s rank correlation test found that *B. choanomphala* abundance positively correlated with calcium, chloride, magnesium, nitrate, phosphate, and potassium levels, but negatively correlated with high water turbulence and increasing pH (abundance decreased with increasing alkalinity). When comparing sites, the Ugandan sites had significantly more *B. choanomphala* snails than the Kenyan and Tanzanian sites. The Ugandan shoreline predominantly had morphotype-B snails, while the Kenyan and Tanzanian shorelines had morphotype-A snails. When categorised by morphotype, the majority of the *B. choanomphala* snails collected from the Ugandan shoreline were morphotype-B, while the majority of the *B. choanomphala* snails collected from the Kenyan and Tanzanian shorelines were morphotype-A. This difference in morphology could be explained by the difference in abiotic factors between the Kenyan, Tanzanian and Ugandan sites, as the Kenyan and Tanzanian sites had significantly higher levels of nitrate, potassium, salinity, sodium, sulphate, TDS and water conductivity than the Ugandan sites. Likewise, morphotype-A abundance had a positive relationship with higher levels of nitrate, potassium, salinity, sodium, sulphate, TDS and water conductivity, while morphotype-B abundance had a negative relationship with higher levels of nitrate, salinity, sodium, sulphate, TDS and water conductivity. Conversely, morphotype-A abundance had a negative relationship with water depth and water turbulence, while morphotype-B abundance had a positive relationship with water depth and water turbulence. The morphotype-A form of *B. choanomphala* was predominately found in shallow and lentic (still) environments, while the morphotype-B form was predominately found in deep lotic (flowing) environments. Dillon (2019) [55] found the American Planorbidae species, *Helisoma trivolvis* also exhibit different ecological phenotypes if they inhabited shallow, lentic waters, or deep, lotic waters. Dillon (2019) [55] hypothesised that these two contrasting shell morphologies helped the snails adapt to their environment as the morphotype found in lentic waters use their shell to trap air and in order to regulate their buoyancy and reach floating vegetation. Conversely, the morphotype found in lotic waters use their wide aperture/foot to grip onto rocks while grazing in flowing water. This functionality could be analogous to the *B. choanomphala* ecophenotypes found in Lake Victoria.

### The factors affecting infection prevalence

A Spearman’s rank correlation test found *S. mansoni* infection in *B. choanomphala* snails had a significant positive relationship with *B. choanomphala* abundance, calcium levels and magnesium levels. Conversely, infection prevalence had a significant negative correlation with pH levels of the lake water (*S. mansoni* infection rate decreased with increasing alkalinity). Rowel et al. (2015) [15] also observed this trend, with *S. mansoni* infection rates having a significant positive relationship with *Biomphalaria* abundance and a significant negative relationship with alkaline pH levels. Previous studies have also found that calcium and magnesium levels correlated with *Biomphalaria* abundances [56,57]. However, *B. choanomphala* abundance itself also has a significant positive relationship with calcium and magnesium levels, as well as a significant negative relationship with alkaline pH levels. Therefore, it is likely that *B. choanomphala* abundance is the only direct factor influencing infection prevalence as the other factors indirectly affect infection via *Biomphalaria* abundance. The highest number of infected snails were found on the Tanzanian shoreline of Lake Victoria. However, no significant differences were found in the number of infected snails across the three shorelines. It is important to state, that other socio-economic, behavioral, or ecological factors not examined in this study may contribute to the higher prevalence of infected *B. choanomphala* when comparing different sites along the shoreline of Lake Victoria. It is also important to note that this analysis does not incorporate human-related data for the sites investigated, which are important when trying to observe patterns of transmission.

### The effects of snail host genetic diversity on infection

Recently, both the genomes of *Biomphalaria pfeifferi* [58] and *B. sudanica*-like [59] snails from the Kenya shoreline of Lake Victoria were sequenced and published. Despite the importance of these species as intermediate hosts of intestinal schistosomiasis at Lake Victoria and the Afrotropical region as a whole, the few number of genomic studies on African *Biomphalaria* species (and other important snail vector species) emphasize how under-researched these vectors are [60].

When we looked at levels of genetic diversity, we found the *B. choanomphala* populations found on the Ugandan shoreline had the highest genetic diversity, followed by the Tanzanian and Kenyan populations. However, the Kenyan populations likely had lowest number of 16S and COI haplotypes due to it being the smallest shoreline when compared to the Ugandan and Tanzanian shorelines. When we compared the level of genetic diversity at sites with and without *S. mansoni* infection, we found sites with infection had a higher mean haplotype diversity score than sites without infection. A Spearman’s rank correlation test found both the 16S and COI Hd scores correlated positively with infection prevalence, but this relationship between haplotype diversity and infection was not statistically significant. When we mapped the distribution of the 16S and COI haplotypes across Lake Victoria, we found infected *B. choanomphala* populations on average had fewer private and more shared 16S and COI haplotypes than *B. choanomphala* populations without infection (Fig 3), indicating there is greater amounts of gene flow occurring among infected sites than among uninfected sites. Our findings are contradictory to previous studies that found a link between lower genetic diversity within a host population and increased susceptibility to parasite infection [10,11,61]. One possible explanation for this contradiction could be due to the higher amounts of gene flow previously mentioned. This is because the migration of *B. choanomphala* snails between sites helps to maintain a high amount of genetic diversity (via gene flow) and can introduce infection into new areas through the arrival of new snails carrying the parasite. This possible movement of snails could explain why sites with infection had higher amounts of genetic diversity compared to sites without infection.

An alternatively explanation for this contradiction could be explained by the ‘co-evolution selective sweep’ phenomenon, where a host-parasite relationship results in selective sweeps of host resistance adaptations and parasite counter-adaptations [62,63]. This causes a reduction in genetic diversity as individuals without this adaptation (e.g., *S. mansoni* resistance) are less successful than those who have it. Populations with high genetic diversity and high *S. mansoni* infection prevalence may not have undergone this selective sweep, while populations with low genetic diversity and low infection prevalence could have. Another explanation could be due to whether non-random mating behaviour is being exhibited or not. This is because non-random mating behaviour is involved in maintaining resistance to *S. mansoni*, and ultimately reduces the genetic diversity of a population [64]. Populations with high genetic diversity and high infection levels may not exhibit non-random mating behaviour, favouring random mating as it promotes genetic diversity over *S. mansoni* resistance, resulting in longer life span, higher fecundity, and more successful offspring [65]. Conversely, populations with low genetic diversity and low infection levels may exhibit this non-random mating behaviour, favouring resistance over genetic diversity. Overall, the relationship between *S. mansoni* and *Biomphalaria* snails is complex and can depend on many factors such as the genetic constitution of the snails, the environment in which they live, and the prevalence and virulence of *S. mansoni* within an area.

In conclusion, the Lake Victoria region is still significantly understudied in terms of the environmental epidemiology of intestinal schistosomiasis. The addition of molecular xenomonitoring infection prevalence data represents a new development and establishes a better ‘infection baseline’ for future snail surveillance studies for understanding *S. mansoni* transmission. Further research is needed to fully explore the relationships between African *Biomphalaria* species, and to elucidate the complex relationship between *Biomphalaria* snails and *S. mansoni* across Lake Victoria.

## Supporting information

S1 Fig Morphological examples of non-lacustrine (morphotype-A) and lacustrine (morphotype-B) forms of *B. choanomphala* snails found at Lake Victoria. The shells are viewed from the apical (left) and umbilical (right) angle.
(TIF)

S2 Fig Examples of habitats surveyed along the shoreline of Lake Victoria, marsh (a), lake edge (b), and other (c). Pictures of site examples are as follows: marshlands (A); lake edge (B); small ditch with standing water in the Busia district, Uganda (C); small stream in the Bugiri district, Uganda (D); and a hybrid of two habitat types, lake edge/marshland hybrid on Kimi Island, Uganda (E).
(TIF)

S3 Fig Maximum likelihood phylogenetic trees of A) gap-free 16S rRNA (330 bp) and B) COI (500 bp) gene fragments. The trees were generated in MEGA v11 using a GTR+Γ model and are rooted on *Biomphalaria glabrata*. The numbers on branches indicate the bootstrap percentages for 1000 replicates (bootstrap values under 50% were not shown) and the scale bar represents 1% sequence divergence.
(TIF)

S1 Table Site information of Lake Victoria collections performed by Standley et al. (2012) [13] and Rowel et al. (2015) [15]. (DOCX)

S2 Table 16S and COI haplotype frequencies and morphology of the sequenced *B. choanomphala* populations by site. (DOCX)

S3 Table Rate of *S. mansoni* infection, haplotype diversity (Hd) scores and nucleotide diversity (π) values of the *B. choanomphala* populations collected at Lake Victoria. (DOCX)

S4 Table The median (IQR) values of the abiotic factors recorded across the Kenyan (*n* = 35), Tanzanian (*n* = 82) and Ugandan (*n* = 53) sites of Lake Victoria. (DOCX)
