# Supplementary figures and images for: Molecular xenomonitoring of Schistosoma mansoni infections in Biomphalaria choanomphala at Lake Victoria, East Africa: Assessing roles of abiotic and biotic factors
Source: PLoS Negl Trop Dis. 2025 Jan 2;19(1):e0012771. doi: 10.1371/journal.pntd.0012771 (PMC11695011; doi:10.1371/journal.pntd.0012771)

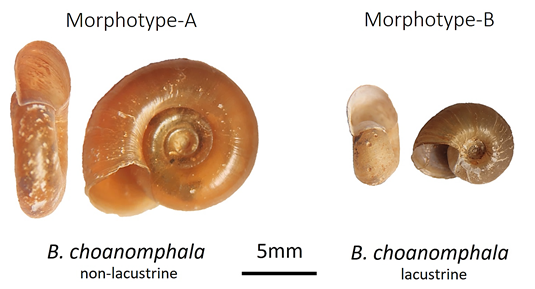

Supplement: S1 Fig — The shells are viewed from the apical (left) and umbilical (right) angle. (TIF) [file pntd.0012771.s001.tif]

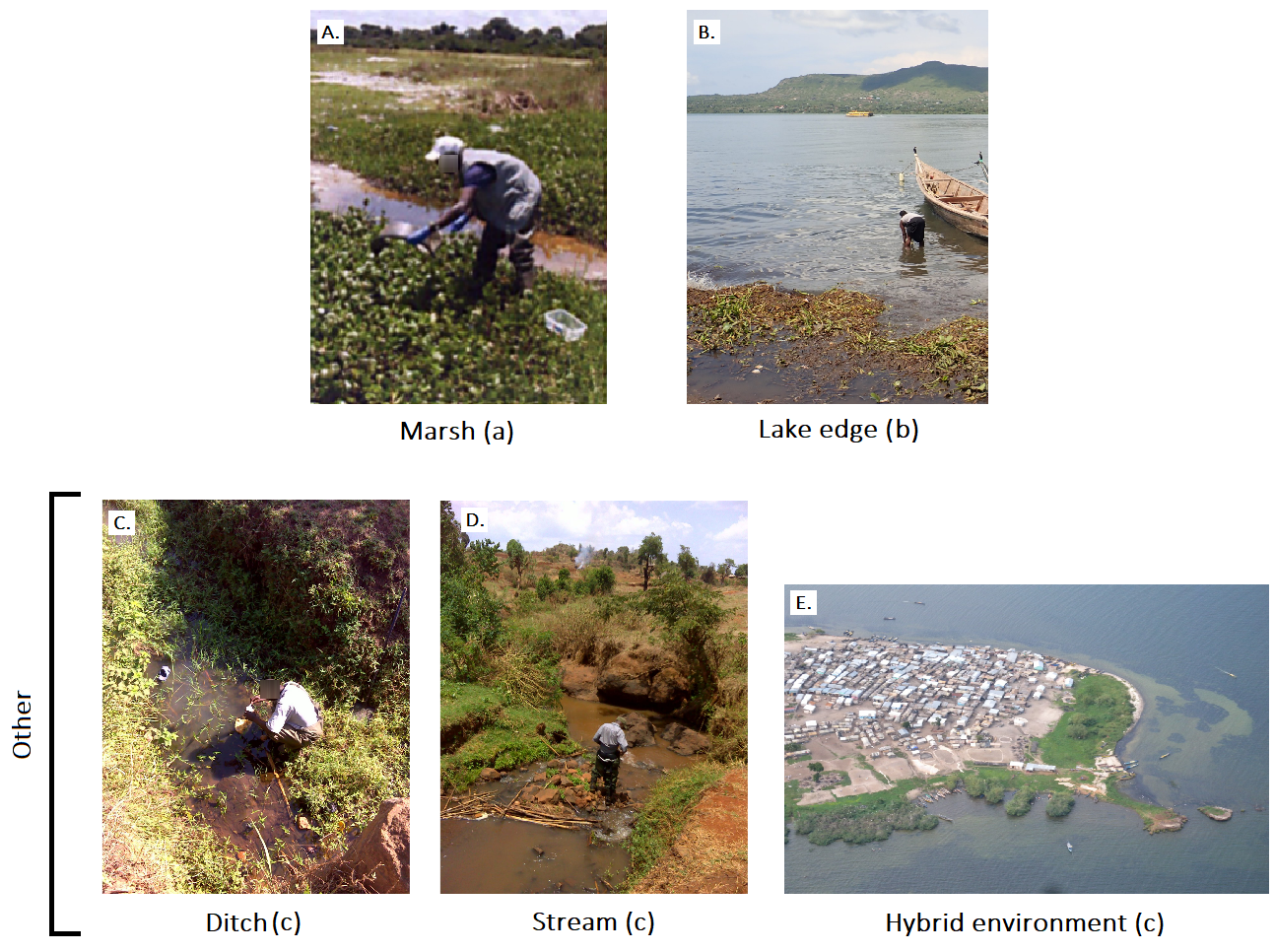

Supplement: S2 Fig — Pictures of site examples are as follows: marshlands (A); lake edge (B); small ditch with standing water in the Busia district, Uganda (C); small stream in the Bugiri district, Uganda (D); and a hybrid of two habitat types, lake edge/marshland hybrid on Kimi Island, Uganda (E). (TIF) [file pntd.0012771.s002.tif]

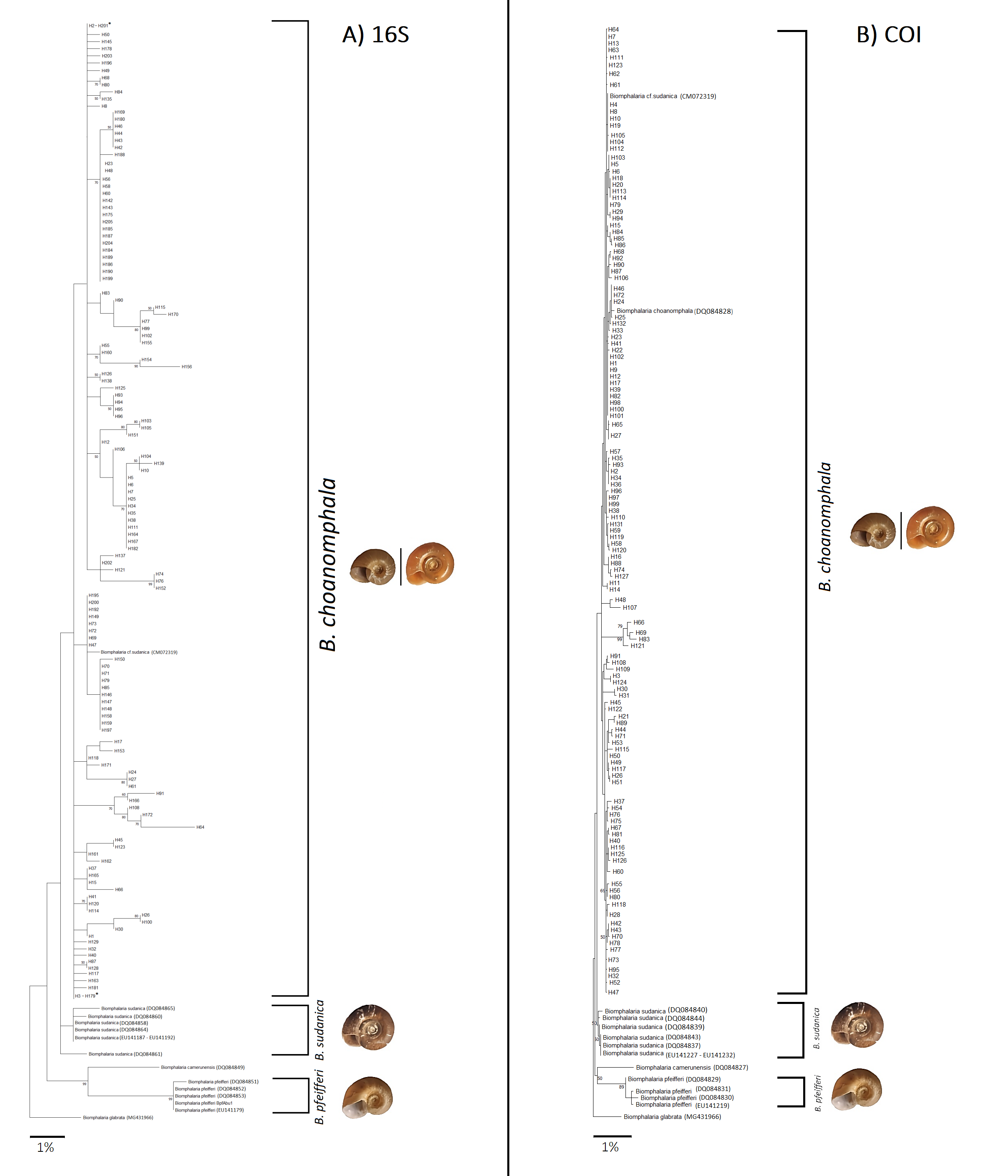

Supplement: S3 Fig — The trees were generated in MEGA v11 using a GTR+Γ model and are rooted on Biomphalaria glabrata. The numbers on branches indicate the bootstrap percentages for 1000 replicates (bootstrap values under 50% were not shown) and the scale bar represents 1% sequence divergence. (TIF) [file pntd.0012771.s003.tif]
