# Supplementary material for: Molecular xenomonitoring of Schistosoma mansoni infections in Biomphalaria choanomphala at Lake Victoria, East Africa: Assessing roles of abiotic and biotic factors
Source: PLoS Negl Trop Dis. 2025 Jan 2;19(1):e0012771. doi: 10.1371/journal.pntd.0012771 (PMC11695011; doi:10.1371/journal.pntd.0012771)
Supplement: S1 Table — (DOCX) [file pntd.0012771.s004.docx]

Supplementary table 1. Site information of Lake Victoria collections performed by Standley et al. (2012) [1] and Rowel et al. (2015) [2].

|  | Extracted  Individuals | Infected | Morphotype | Latitude | Longitude |
| --- | --- | --- | --- | --- | --- |
| K001a | 11 | 2 | A | - 0.073 | 34.058 |
| K001b | 0 | 0 | - | - 0.073 | 34.058 |
| K002a | 11 | 1 | A | - 0.110 | 34.065 |
| K003a | 4 | 0 | A | - 0.187 | 34.387 |
| K003b | 0 | 0 | - | - 0.187 | 34.387 |
| K004b | 4 | 2 | A | - 0.141 | 34.594 |
| K005c (Lake/Marsh) | 5 | 0 | A | - 0.420 | 34.207 |
| K006a | 10 | 0 | A | - 0.434 | 34.171 |
| K007a | 4 | 0 | A | - 0.431 | 34.129 |
| K007b | 0 | 0 | - | - 0.431 | 34.129 |
| K008a | 8 | 0 | A | - 0.398 | 34.160 |
| K010b | 0 | 0 | - | - 0.381 | 34.213 |
| K011c (Lake/Marsh) | 4 | 0 | A | - 0.453 | 34.323 |
| K012b | 2 | 0 | A | - 0.474 | 34.288 |
| Κ013a | 0 | 0 | - | - 1.010 | 34.130 |
| K013b | 12 | 0 | B | - 1.010 | 34.130 |
| K014b | 0 | 0 | - | - 1.001 | 34.099 |
| K015a | 5 | 0 | A | - 0.856 | 34.187 |
| Κ016b | 0 | 0 | - | - 0.817 | 34.119 |
| K017a | 4 | 0 | A | - 0.726 | 34.058 |
| Κ018c (Lake/Marsh) | 0 | 0 | - | - 0.538 | 34.164 |
| K019a | 6 | 0 | A | - 0.437 | 34.015 |
| K020b | 11 | 0 | A | - 0.523 | 34.455 |
| K021a | 4 | 0 | A | - 0.354 | 34.663 |
| Κ021b | 0 | 0 | - | - 0.354 | 34.663 |
| K022c (Ditch/Marsh) | 2 | 0 | A | - 0.312 | 34.848 |
| Κ023c (Rice Paddy) | 0 | 0 | - | - 0.226 | 34.967 |
| K024c (Pond) | 0 | 0 | - | - 0.175 | 34.936 |
| K025c (Pond/Paddy) | 0 | 0 | - | - 0.171 | 34.907 |
| K026c (Pond) | 0 | 0 | - | - 0.156 | 34.850 |
| K028b | 2 | 1 | A | - 0.105 | 34.718 |
| K029b | 12 | 0 | A | - 0.096 | 34.749 |
| K030b | 3 | 0 | B | - 0.328 | 34.267 |
| K032c (Pond) | 0 | 0 | - | - 0.181 | 34.263 |
| K033a | 5 | 0 | A | 0.099 | 33.968 |
| T001c (Lake/Marsh) | 11 | 1 | Both | - 2.713 | 32.894 |
| T002a | 6 | 0 | A | - 2.726 | 32.870 |
| T003c (Pond) | 5 | 0 | A | - 2.643 | 32.960 |
| T004c (Lake/Marsh) | 4 | 1 | Both | - 2.531 | 32.901 |
| T005c (Rice Paddy) | 4 | 0 | A | - 2.414 | 32.941 |
| T006a | 8 | 1 | A | - 2.405 | 32.950 |
| T006b | 0 | 0 | - | - 2.405 | 32.950 |
| T007b | 0 | 0 | - | - 2.502 | 32.880 |
| T008c (Pond) | 0 | 0 | - | - 2.585 | 33.390 |
| T009a | 3 | 0 | A | - 2.525 | 33.395 |
| T009b | 0 | 0 | - | - 2.526 | 33.395 |
| T010c (Pond) | 0 | 0 | - | - 2.588 | 33.415 |
| T011b | 11 | 0 | B | - 2.452 | 33.517 |
| T012a | 0 | 0 | - | - 2.262 | 33.809 |
| T013b | 0 | 0 | - | - 2.168 | 33.351 |
| T014a | 0 | 0 | - | - 2.112 | 33.210 |
| T014b | 5 | 2 | B | - 2.112 | 33.210 |
| T015a | 4 | 1 | A | - 2.128 | 33.048 |
| T016a | 11 | 0 | A | - 2.117 | 33.071 |
| T017b | 4 | 0 | B | - 2.124 | 33.065 |
| T018a | 4 | 1 | B | - 2.018 | 33.103 |
| T019c (Lake/Marsh) | 3 | 0 | A | - 1.996 | 33.111 |
| T020a | 4 | 0 | A | - 1.984 | 33.018 |
| T021a | 4 | 0 | A | - 1.943 | 32.861 |
| T022a | 3 | 0 | A | - 2.048 | 33.312 |
| T023a | 4 | 1 | A | - 2.131 | 33.328 |
| T024a | 0 | 0 | - | - 2.157 | 33.477 |
| T025c (Marsh/Field) | 3 | 1 | A | - 2.080 | 33.742 |
| T026a | 11 | 2 | A | - 2.535 | 32.755 |
| T027b | 10 | 7 | B | - 2.546 | 32.542 |
| T028c (Rice Paddy/Ditch) | 0 | 0 | - | - 2.538 | 32.233 |
| T029a | 1 | 1 | B | - 2.507 | 32.015 |
| T030b | 5 | 0 | B | - 2.494 | 31.986 |
| T031b | 5 | 0 | A | - 2.461 | 31.984 |
| T032b | 1 | 0 | A | - 2.440 | 32.010 |
| T033a | 10 | 4 | A | - 2.405 | 32.059 |
| T034a | 4 | 1 | A | - 2.348 | 32.040 |
| T035b | 0 | 0 | - | - 2.383 | 31.967 |
| T036a | 11 | 2 | A | - 2.407 | 31.945 |
| T037b | 4 | 2 | B | - 2.415 | 31.923 |
| T038a | 3 | 0 | A | - 2.436 | 32.411 |
| T038b | 0 | 0 | - | - 2.436 | 32.411 |
| T039c (Lake/Marsh) | 0 | 0 | - | - 2.539 | 32.840 |
| T040c (Lake/Marsh) | 10 | 0 | A | - 2.528 | 32.895 |
| T041a | 0 | 0 | - | - 1.962 | 33.530 |
| T042a | 4 | 0 | A | - 1.956 | 33.467 |
| T042b | 0 | 0 | - | - 1.956 | 33.467 |
| T043a | 2 | 1 | A | - 2.047 | 33.381 |
| T043b | 0 | 0 | - | - 2.047 | 33.381 |
| T043c (Marsh/Field) | 0 | 0 | - | - 2.047 | 33.381 |
| T044b | 2 | 0 | B | - 2.015 | 33.386 |
| T044c (Marsh/Field) | 0 | 0 | - | - 2.015 | 33.386 |
| T045b | 2 | 0 | A | - 1.984 | 33.433 |
| T047a | 4 | 0 | A | - 1.809 | 33.412 |
| T047b | 0 | 0 | - | - 1.809 | 33.412 |
| T048a | 3 | 0 | A | - 1.908 | 33.397 |
| T048b | 0 | 0 | - | - 1.908 | 33.397 |
| T049b | 0 | 0 | - | - 1.847 | 33.466 |
| T050a | 0 | 0 | - | - 1.782 | 33.622 |
| T050b | 0 | 0 | - | - 1.782 | 33.622 |
| T051b | 0 | 0 | - | - 1.680 | 33.541 |
| T052b | 0 | 0 | - | - 1.677 | 33.618 |
| T053a | 0 | 0 | - | - 1.683 | 33.687 |
| T053b | 0 | 0 | - | - 1.683 | 33.687 |
| T054b | 0 | 0 | - | - 1.605 | 33.695 |
| T056b | 0 | 0 | - | - 1.496 | 33.739 |
| T057b | 4 | 0 | B | - 1.330 | 33.813 |
| T059b | 0 | 0 | - | - 1.454 | 33.856 |
| T060a | 0 | 0 | - | - 1.498 | 33.895 |
| T060b | 0 | 0 | - | - 1.498 | 33.895 |
| T061c (Pond) | 4 | 1 | B | - 1.599 | 33.913 |
| T062c (Ditch/Pond) | 0 | 0 | - | - 1.516 | 33.821 |
| T063c (Ditch/Pond) | 4 | 0 | A | - 1.526 | 33.832 |
| T064a | 10 | 0 | A | - 1.347 | 33.970 |
| T064b | 0 | 0 | - | - 1.347 | 33.970 |
| T065c (Pond) | 0 | 0 | - | - 1.401 | 34.134 |
| T066b | 5 | 0 | B | - 1.306 | 33.955 |
| T067a | 4 | 0 | A | - 1.125 | 33.999 |
| T068b | 9 | 1 | B | - 1.038 | 34.085 |
| T069b | 0 | 0 | - | - 1.193 | 33.943 |
| T070a | 4 | 0 | A | - 1.255 | 33.868 |
| T070b | 0 | 0 | - | - 1.255 | 33.868 |
| U001b | 3 | 0 | B | 0.078 | 32.448 |
| U002b | 0 | 0 | - | - 0.234 | 32.575 |
| U003b | 0 | 0 | - | - 0.352 | 32.572 |
| U004c (Lake/Stream) | 0 | 0 | - | - 0.320 | 32.576 |
| U005b | 11 | 0 | B | - 0.364 | 32.295 |
| U006a | 2 | 0 | B | - 0.334 | 32.332 |
| U007c (Lake/Marsh) | 0 | 0 | - | - 0.325 | 32.309 |
| U008b | 4 | 0 | B | - 0.511 | 32.158 |
| U009b | 1 | 0 | B | - 0.310 | 32.292 |
| U010b | 1 | 1 | B | - 0.324 | 32.194 |
| U011b | 2 | 1 | B | - 0.248 | 32.068 |
| U012a | 10 | 0 | Both | - 0.273 | 32.027 |
| U013b | 1 | 1 | B | - 0.301 | 32.035 |
| U014b | 2 | 0 | B | 0.004 | 32.432 |
| U015b | 2 | 0 | B | 0.015 | 32.388 |
| U016b | 4 | 0 | B | 0.016 | 32.381 |
| U017b | 0 | 0 | - | - 0.009 | 32.432 |
| U019b | 0 | 0 | - | - 0.915 | 31.767 |
| U020a | 11 | 0 | A | - 0.939 | 31.763 |
| U021a | 10 | 0 | A | - 0.655 | 31.797 |
| U022b | 0 | 0 | - | - 0.348 | 31.880 |
| U023a | 11 | 0 | Both | 0.015 | 32.767 |
| U024b | 10 | 1 | B | - 0.042 | 32.764 |
| U025b | 7 | 1 | B | 0.002 | 32.901 |
| U026b | 1 | 0 | B | - 0.110 | 32.764 |
| U027a | 3 | 2 | B | - 0.100 | 32.653 |
| U028b | 11 | 0 | B | - 0.086 | 32.652 |
| U029b | 8 | 0 | B | 0.141 | 33.602 |
| U030b | 11 | 2 | B | 0.112 | 33.602 |
| U034b | 2 | 0 | B | 0.003 | 33.659 |
| U035b | 9 | 1 | B | 0.156 | 33.566 |
| U036b | 1 | 0 | B | 0.173 | 33.562 |
| U037c (Lake/Marsh) | 11 | 1 | Both | 0.318 | 33.627 |
| U038a | 0 | 0 | - | 0.263 | 33.985 |
| U038b | 3 | 1 | A | 0.263 | 33.985 |
| U039a | 4 | 2 | Both | 0.253 | 33.989 |
| U040b | 4 | 2 | B | 0.241 | 33.992 |
| U041a | 0 | 0 | - | 0.535 | 33.891 |
| U043c (Lake/Marsh) | 0 | 0 | - | 0.476 | 33.281 |
| U044b | 4 | 0 | Both | 0.438 | 33.241 |
| U045b | 0 | 0 | - | 0.290 | 32.655 |
| U046b | 11 | 0 | B | 0.173 | 33.184 |
| U047b | 4 | 0 | B | 0.186 | 33.215 |
| U048b | 4 | 0 | B | 0.198 | 33.265 |
| U049b | 4 | 0 | B | 0.234 | 33.243 |
| U050c (Lake/Marsh) | 4 | 0 | Both | 0.247 | 33.219 |
| U051b | 0 | 0 | - | 0.270 | 33.206 |
| U052b | 3 | 0 | B | 0.271 | 33.153 |
| U053b | 8 | 2 | B | 0.240 | 33.137 |
| U055b | 7 | 0 | B | - 0.092 | 32.684 |
| Bugoto-c (Lake/Marsh) | 20 | 1 | Both | 0.319 | 33.620 |
| Bukoba-c (Lake/Marsh) | 20 | 1 | Both | 0.312 | 33.492 |
| Lwanika-c (Lake/Marsh) | 20 | 2 | Both | 0.351 | 33.446 |

Note: (a), (b) and (c) indicates whether the collection site was either (a) marshlands, (b) the lake edge or (c) other (e.g. ditch, hybrid environment etc.). Sites that had samples genotyped using the 16S and COI gene fragments are highlighted. ‘Both’ indicates both morphotype-A and B snails were recorded.

References:

1. Standley CJ, Vounatsou P, Gosoniu L, Jorgensen A, Adriko M, Lwambo NJS, et al. The distribution of Biomphalaria (Gastropoda: Planorbidae) in Lake Victoria with ecological and spatial predictions using Bayesian modelling. Hydrobiologia. 2012;683(1):249-64.
2. Rowel C, Fred B, Betson M, Sousa-Figueiredo JC, Kabatereine NB, Stothard JR. Environmental epidemiology of intestinal schistosomiasis in Uganda: population dynamics of Biomphalaria (Gastropoda: Planorbidae) in Lake Albert and Lake Victoria with observations on natural infections with digenetic trematodes. BioMed research international. 2015;2015.
