# Supplementary material for: Molecular xenomonitoring of Schistosoma mansoni infections in Biomphalaria choanomphala at Lake Victoria, East Africa: Assessing roles of abiotic and biotic factors
Source: PLoS Negl Trop Dis. 2025 Jan 2;19(1):e0012771. doi: 10.1371/journal.pntd.0012771 (PMC11695011; doi:10.1371/journal.pntd.0012771)
Supplement: S2 Table — (DOCX) [file pntd.0012771.s005.docx]

Supplementary table 2. 16S and COI haplotype frequencies and morphology of the sequenced *B.* *choanomphala* populations by site.

| Site | 16S | | | COI | | |
| --- | --- | --- | --- | --- | --- | --- |
|  | Haplotype [GB Accession] | Morphotype | Frequency | Haplotype [GB Accession] | Morphotype | Frequency |
| K001a | **69** [HM769018] | A | 5 | 4 [HM769135] | A | 4 |
|  | 70  [HM769019] | A | 3 | **5** [HM769136] | A | 4 |
|  | 71  [HM769020] | A | 2 | 6 [HM769137] | A | 1 |
|  | 147  [HM769096] | A | 1 | 104 [HM769235] | A | 1 |
| K002a | 68 [HM769017] | A | 2 | **5** [HM769135] | A | 5 |
|  | 70 [HM769019] | A | 1 | **7** [HM769138] | A | 1 |
|  | 72  [HM769021] | A | 1 | **8** [HM769139] | A | 1 |
|  | 73  [HM769022] | A | 1 | 10 [HM769141] | A | 1 |
|  | 75  [HM769024] | A | 1 | 13 [HM769144] | A | 1 |
|  | 85  [HM769034] | A | 1 | 105 [HM769236] | A | 1 |
|  | 148  [HM769097] | A | 1 |  |  |  |
|  | 149  [HM769098] | A | 1 |  |  |  |
|  | 150  [HM769099] | A | 1 |  |  |  |
| K006a | **79** [HM769028] | A | 6 | 9 [HM769140] | A | 3 |
|  | 80 [HM769029] | A | 3 | 20 [HM769151] | A | 2 |
|  |  |  |  | 113 [HM769244] | A | 4 |
|  |  |  |  | 114 [HM769245] | A | 1 |
| K013b | **51** [HM769000] | B | 2 | **1** [HM769132] | B | 2 |
|  | 78 [HM769027] | B | 4 | **5** [HM769136] | B | 1 |
|  | **79** [HM769028] | B | 1 | 12 [HM769143] | B | 2 |
|  | 157 [HM769106] | B | 2 | 15 [HM769146] | B | 2 |
|  | 175 [HM769124] | B | 1 | 17 [HM769148] | B | 1 |
|  | 176 [HM769125] | B | 1 | 110 [HM769241] | B | 2 |
|  | 177 [HM769126] | B | 1 | 111 [HM769242] | B | 1 |
| K020b | **69** [HM769018] | A | 5 | **5** [HM769136] | A | 4 |
|  | 70 [HM769019] | A | 1 | **8** [HM769139] | A | 3 |
|  | **79** [HM769028] | A | 1 | 18 [HM769149] | A | 1 |
|  | 158 [HM769107] | A | 1 | 19 [HM769150] | A | 1 |
|  | 159 [HM769108] | A | 2 | **61** [HM769192] | A | 1 |
|  | 160 [HM769109] | A | 1 | 112 [HM769243] | A | 1 |
| K029b | **69** [HM769018] | A | 6 | **5** [HM769136] | A | 3 |
|  | **79** [HM769028] | A | 1 | **7** [HM769138] | A | 2 |
|  | 85 [HM769034] | A | 1 | **8** [HM769139] | A | 6 |
|  | 146 [HM769095] | A | 1 | 103 [HM769234] | A | 1 |
| T001c | 8 [HM768957] | A | 1 | **1** [HM769132] | Both | 7 |
|  | **9** [HM768958] | Both | 4 | **7** [HM769138] | B | 1 |
|  | 67 [HM769016] | A | 1 | 24 [HM769155] | A | 1 |
|  | 89 [HM769038] | A | 1 | 82 [HM769213] | A | 1 |
|  | 90 [HM769039] | A | 1 | 83 [HM769214] | A | 1 |
|  | 91 [HM769040] | A | 1 |  |  |  |
|  | 92 [HM769041] | B | 1 |  |  |  |
| T011b | **9** [HM768958] | B | 1 | 21 [HM769152] | B | 2 |
|  | 29 [HM768978] | B | 1 | 22 [HM769153] | B | 1 |
|  | 30 [HM768979] | B | 1 | **23** [HM769154] | B | 2 |
|  | **51** [HM769000] | B | 1 | 84 [HM769215] | B | 4 |
|  | 93 [HM769042] | B | 3 | 85 [HM769216] | B | 1 |
|  | 94 [HM769043] | B | 1 | 86 [HM769217] | B | 1 |
|  | 95 [HM769044] | B | 1 |  |  |  |
|  | 96 [HM769045] | B | 1 |  |  |  |
| T016a | **9** [HM768958] | A | 3 | **1** [HM769132] | A | 4 |
|  | 10 [HM768959] | A | 4 | **23** [HM769154] | A | 3 |
|  | 11 [HM768960] | A | 4 | 24 [HM769155] | A | 4 |
| T026a | **9** [HM768958] | A | 2 | **1** [HM769132] | A | 1 |
|  | 11 [HM768960] | A | 3 | 24 [HM769155] | A | 8 |
|  | 12 [HM768961] | A | 1 | 25 [HM769156] | A | 1 |
|  | 19 [HM768968] | A | 2 | 87 [HM769218] | A | 1 |
|  | **65** [HM769014] | A | 1 |  |  |  |
|  | 97 [HM769046] | A | 2 |  |  |  |
| T027b | 24 [HM768973] | B | 1 | 30 [HM769161] | B | 2 |
|  | 26 [HM768975] | B | 1 | 31 [HM769162] | B | 1 |
|  | 27 [HM768976] | B | 1 | 88 [HM769219] | B | 3 |
|  | 61 [HM769010] | B | 1 | 89 [HM769220] | B | 2 |
|  | 99 [HM769048] | B | 1 | 90 [HM769221] | B | 1 |
|  | 100 [HM769049] | B | 2 | 91 [HM769222] | B | 1 |
|  | 101 [HM769050] | B | 1 |  |  |  |
|  | 102 [HM769051] | B | 2 |  |  |  |
| T033a | 25 [HM768974] | A | 1 | 32 [HM769163] | A | 5 |
|  | 28 [HM768977] | A | 1 | 33 [HM769164] | A | 3 |
|  | 62 [HM769011] | A | 5 | 92 [HM769223] | A | 1 |
|  | 84 [HM769033] | A | 3 |  |  |  |
| T036a | 31 [HM768980] | A | 5 | **5** [HM769136] | A | 2 |
|  | 134 [HM769083] | A | 1 | 29 [HM769160] | A | 5 |
|  | 135 [HM769084] | A | 1 | 94 [HM769225] | A | 1 |
|  | 136 [HM769085] | A | 1 | 95 [HM769226] | A | 1 |
|  | 137 [HM769086] | A | 1 | 96 [HM769227] | A | 1 |
|  | 138 [HM769087] | A | 1 |  |  |  |
|  | 139 [HM769088] | A | 1 |  |  |  |
| T040c | **9** [HM768958] | A | 2 | **1** [HM769132] | A | 3 |
|  | 23 [HM768972] | A | 1 | 38 [HM769169] | A | 1 |
|  | 57 [HM769006] | A | 1 | 97 [HM769228] | A | 1 |
|  | 140 [HM769089] | A | 1 | 98 [HM769229] | A | 1 |
|  | 141 [HM769090] | A | 1 | 99 [HM769230] | A | 1 |
|  | 142 [HM769091] | A | 1 | 100 [HM769231] | A | 1 |
|  | 143 [HM769092] | A | 1 | 101 [HM769232] | A | 1 |
|  | 144 [HM769093] | A | 1 | 123 [HM769254] | A | 1 |
|  | 174 [HM769123] | A | 1 |  |  |  |
| T064a | **51** [HM769000] | A | 1 | **1** [HM769132] | A | 1 |
|  | **65** [HM769014] | A | 2 | 3 [HM769134] | A | 2 |
|  | 66 [HM769015] | A | 3 | 9 [HM769140] | A | 4 |
|  | 67 [HM769016] | A | 2 | 98 [HM769229] | A | 1 |
|  | 68 [HM769017] | A | 1 | 102 [HM769233] | A | 1 |
|  | 145 [HM769094] | A | 1 | 124 [HM769255] | A | 1 |
| U005b | 4 [HM768953] | B | 2 | **1** [HM769132] | B | 1 |
|  | 5 [HM768954] | B | 1 | 32 [HM769163] | B | 1 |
|  | 33 [HM768982] | B | 1 | 39 [HM769170] | B | 1 |
|  | 103 [HM769052] | B | 1 | 40 [HM769171] | B | 5 |
|  | 104 [HM769053] | B | 1 | 41 [HM769172] | B | 1 |
|  | 105 [HM769054] | B | 1 | 68 [HM769199] | B | 1 |
|  | 106 [HM769055] | B | 1 | 69 [HM769200] | B | 1 |
|  | 107 [HM769056] | B | 1 |  |  |  |
|  | 108 [HM769057] | B | 1 |  |  |  |
|  | 109 [HM769058] | B | 1 |  |  |  |
| U012a | 6 [HM768955] | A | 1 | **1** [HM769132] | A | 1 |
|  | 7 [HM768956] | A | 1 | **42** [HM769173] | Both | 6 |
|  | **9** [HM768958] | A | 1 | 43 [HM769174] | A | 1 |
|  | 34 [HM768983] | A | 1 | 70 [HM769201] | A | 2 |
|  | 35 [HM768984] | A | 1 |  |  |  |
|  | **110** [HM769059] | Both | 2 |  |  |  |
|  | **111** [HM769060] | Both | 3 |  |  |  |
| U020a | 21 [HM768970] | A | 1 | 44 [HM769175] | A | 1 |
|  | 36 [HM768985] | A | 1 | 45 [HM769176] | A | 9 |
|  | 112 [HM769061] | A | 6 | 71 [HM769202] | A | 1 |
|  | 113 [HM769062] | A | 1 |  |  |  |
| U021a | 19 [HM768968] | A | 8 | 46 [HM769177] | A | 9 |
|  | 20 [HM768969] | A | 1 | 72 [HM769203] | A | 1 |
|  | 83 [HM769032] | A | 1 |  |  |  |
| U023a | **17** [HM768966] | Both | 2 | 47 [HM769178] | A | 1 |
|  | 18 [HM768967] | A | 2 | **48** [HM769179] | Both | 2 |
|  | 37 [HM768986] | A | 1 | 49 [HM769180] | A | 1 |
|  | 38 [HM768987] | B | 1 | 50 [HM769181] | B | 1 |
|  | 161 [HM769110] | B | 1 | 52 [HM769183] | A | 1 |
|  | 162 [HM769111] | B | 1 | 115 [HM769246] | B | 1 |
|  | 163 [HM769112] | B | 1 | 116 [HM769247] | A | 1 |
|  | 164 [HM769113] | A | 1 | 125 [HM769256] | B | 1 |
|  | 179 [HM769128] | A | 1 | 126 [HM769257] | B | 1 |
| U028b | 40 [HM768989] | B | 1 | 54 [HM769185] | B | 1 |
|  | 41 [HM768990] | B | 1 | 55 [HM769186] | B | 1 |
|  | 53 [HM769002] | B | 1 | 56 [HM769187] | B | 3 |
|  | 114 [HM769063] | B | 1 | 73 [HM769204] | B | 1 |
|  | 115 [HM769064] | B | 1 | 74 [HM769205] | B | 1 |
|  | 116 [HM769065] | B | 1 | 75 [HM769206] | B | 1 |
|  | 117 [HM769066] | B | 1 | 76 [HM769207] | B | 2 |
|  | 118 [HM769067] | B | 1 | 77 [HM769208] | B | 1 |
|  | 119 [HM769068] | B | 1 |  |  |  |
|  | 120 [HM769069] | B | 1 |  |  |  |
| U030b | 42 [HM768991] | B | 1 | 32 [HM769163] | B | 1 |
|  | 43 [HM768992] | B | 1 | 57 [HM769188] | B | 2 |
|  | 44 [HM768993] | B | 1 | 58 [HM769189] | B | 1 |
|  | 46 [HM768995] | B | 2 | 59 [HM769190] | B | 2 |
|  | 169 [HM769118] | B | 1 | 119 [HM769250] | B | 1 |
|  | 170 [HM769119] | B | 1 | 120 [HM769251] | B | 1 |
|  | 171 [HM769120] | B | 1 | 121 [HM769252] | B | 1 |
|  | 172 [HM769121] | B | 1 | 122 [HM769253] | B | 1 |
|  | 173 [HM769122] | B | 1 | 127 [HM769258] | B | 1 |
|  | 180 [HM769129] | B | 1 |  |  |  |
| U037c | 2 [HM768951] | B | 1 | **8** [HM769139] | B | 1 |
|  | 47 [HM768996] | B | 1 | **27** [HM769158] | B | 1 |
|  | **48** [HM768997] | B | 1 | 60 [HM769191] | B | 1 |
|  | 54 [HM769003] | B | 1 | **61** [HM769192] | B | 1 |
|  | 55 [HM769004] | B | 1 | 62 [HM769193] | B | 2 |
|  | 56 [HM769005] | B | 1 | 63 [HM769194] | B | 1 |
|  | 58 [HM769007] | B | 1 | **64** [HM769195] | Both | 2 |
|  | **60** [HM769009] | Both | 2 | 65 [HM769196] | B | 1 |
|  | 121 [HM769070] | B | 1 | 78 [HM769209] | B | 1 |
| U046b | 3 [HM768952] | B | 1 | 32 [HM769163] | B | 2 |
|  | 64 [HM769013] | B | 1 | 66 [HM769197] | B | 1 |
|  | 86 [HM769035] | B | 1 | 67 [HM769198] | B | 5 |
|  | 87 [HM769036] | B | 1 | 80 [HM769211] | B | 2 |
|  | 88 [HM769037] | B | 1 | 81 [HM769212] | B | 1 |
|  | 124 [HM769073] | B | 1 |  |  |  |
|  | 128 [HM769077] | B | 1 |  |  |  |
|  | 129 [HM769078] | B | 1 |  |  |  |
|  | 130 [HM769079] | B | 1 |  |  |  |
| Bugoto c | **48** [HM768997] | Both | 6 | **27** [HM769158] | Both | 6 |
|  | **60** [HM769009] | Both | 4 | **61** [HM769192] | B | 1 |
|  | 126 [HM769075] | B | 1 | 62 [HM769193] | B | 7 |
|  | 127 [HM769076] | B | 2 | **64** [HM769195] | Both | 4 |
|  | 160 [HM769109] | B | 1 | 128 [OQ849943] | B | 2 |
|  | 185 [[OQ924888](https://www.ncbi.nlm.nih.gov/nucleotide/OQ924888.1?report=genbank&log$=nucltop&blast_rank=4&RID=8UKJKGAR016)] | B | 1 |  |  |  |
|  | 187 [OQ924869] | B | 1 |  |  |  |
|  | 202 [OQ924871] | B | 1 |  |  |  |
|  | 203 [OQ924872] | B | 1 |  |  |  |
|  | 204 [OQ924876] | B | 1 |  |  |  |
|  | 205 [OQ924882] | B | 1 |  |  |  |
| Bukoba c | 42 [HM768991] | B | 1 | **27** [HM769158] | Both | 4 |
|  | **48** [HM768997] | Both | 4 | **61** [HM769192] | B | 2 |
|  | **60** [HM769009] | B | 2 | 62 [HM769193] | B | 5 |
|  | 160 [HM769109] | B | 1 | 63 [HM769194] | B | 1 |
|  | 183 [[OQ924898](https://www.ncbi.nlm.nih.gov/nucleotide/OQ924898.1?report=genbank&log$=nucltop&blast_rank=2&RID=8UEC4YFU016)] | B | 1 | **64** [HM769195] | B | 3 |
|  | 184 [OQ924907] | B | 2 | 128 [OQ849965] | B | 1 |
|  | 185 [[OQ924908](https://www.ncbi.nlm.nih.gov/nucleotide/OQ924908.1?report=genbank&log$=nucltop&blast_rank=2&RID=8UKJKGAR016)] | B | 1 | 129 [OQ849957] | B | 1 |
|  | 186 [OQ924883] | B | 1 | 130 [OQ849967] | B | 1 |
|  | 188 [OQ924889] | B | 1 | 131 [OQ849969] | B | 1 |
|  | 189 [OQ924890] | B | 1 | 132 [OQ849973] | B | 1 |
|  | 190 [OQ924891] | B | 1 |  |  |  |
|  | 191 [OQ924892] | B | 1 |  |  |  |
|  | 192 [OQ924901] | B | 1 |  |  |  |
|  | 193 [OQ924902] | B | 1 |  |  |  |
|  | 194 [OQ924905] | B | 1 |  |  |  |
| Lwanika c | **48** [HM768997] | B | 1 | **8** [HM769139] | B | 3 |
|  | **60** [HM769009] | B | 1 | **27** [HM769158] | Both | 8 |
|  | **65** [HM769014] | B | 1 | 46 [HM769177] | B | 1 |
|  | **69** [HM769018] | B | 2 | 62 [HM769193] | B | 1 |
|  | 127 [[HM769076](https://www.ncbi.nlm.nih.gov/nuccore/HM769076.1)] | B | 1 | 63 [HM769194] | B | 2 |
|  | **183** [OQ924918] | Both | 4 | **64** [HM769195] | B | 2 |
|  | 184 [[OQ924927](https://www.ncbi.nlm.nih.gov/nucleotide/OQ924927.1?report=genbank&log$=nucltop&blast_rank=1&RID=8UKJDYFR016)] | B | 1 | 79 [HM769210] | B | 1 |
|  | 185 [[OQ924928](https://www.ncbi.nlm.nih.gov/nucleotide/OQ924928.1?report=genbank&log$=nucltop&blast_rank=1&RID=8UKJKGAR016)] | B | 1 | 132 [OQ849990] | B | 1 |
|  | 186 [[OQ924915](https://www.ncbi.nlm.nih.gov/nucleotide/OQ924915.1?report=genbank&log$=nucltop&blast_rank=2&RID=8UJ8EKVC016)] | B | 1 | 133 [OQ849987] | B | 1 |
|  | 195 [OQ924911] | B | 1 |  |  |  |
|  | 196 [OQ924913] | B | 1 |  |  |  |
|  | 197 [OQ924914] | B | 1 |  |  |  |
|  | 198 [OQ924917] | B | 1 |  |  |  |
|  | 199 [OQ924919] | B | 1 |  |  |  |
|  | 200 [OQ924921] | B | 1 |  |  |  |
|  | 201 [OQ924924] | B | 1 |  |  |  |

Note: sites with *S. mansoni* infection present are highlighted. ‘Both’ indicates both morphotype A and B snails were recorded. Haplotypes that exhibited both morphotypes are in bold.
