# Supplementary material for: Molecular xenomonitoring of Schistosoma mansoni infections in Biomphalaria choanomphala at Lake Victoria, East Africa: Assessing roles of abiotic and biotic factors
Source: PLoS Negl Trop Dis. 2025 Jan 2;19(1):e0012771. doi: 10.1371/journal.pntd.0012771 (PMC11695011; doi:10.1371/journal.pntd.0012771)
Supplement: S3 Table — (DOCX) [file pntd.0012771.s006.docx]

Supplementary table 3. The rate of *S. mansoni* infection, haplotype diversity (Hd) scores and nucleotide diversity (π) values of the *B. choanomphala* populations collected at Lake Victoria.

|  | Site | No. | Infected | 16S | | COI | |
| --- | --- | --- | --- | --- | --- | --- | --- |
|  |  |  |  | Hd | π | Hd | π |
| Kenya | K001a | 11 | 2 | 0.746 | 0.005 | 0.836 | 0.005 |
|  | K002a | 11 | 1 | 0.978 | 0.011 | 0.712 | 0.001 |
|  | K004b | 4 | 2 | - | - | - | - |
|  | K006a | 10 | 0 | 0.5 | 0.005 | 0.855 | 0.014 |
|  | K013b | 12 | 0 | 0.879 | 0.012 | 0.909 | 0.014 |
|  | K020b | 11 | 0 | 0.8 | 0.006 | 0.933 | 0.004 |
|  | K028b | 2 | 1 | - | - | - | - |
|  | K029b | 12 | 0 | 0.583 | 0.004 | 0.818 | 0.015 |
| Tanzania | T001c | 11 | 1 | 0.867 | 0.017 | 0.978 | 0.017 |
|  | T004c | 4 | 1 | - | - | - | - |
|  | T006a | 8 | 1 | - | - | - | - |
|  | T011b | 11 | 0 | 0.933 | 0.016 | 0.927 | 0.009 |
|  | T014b | 5 | 2 | - | - | - | - |
|  | T016a | 11 | 1 | 0.727 | 0.008 | 0.964 | 0.016 |
|  | T018a | 4 | 1 | - | - | - | - |
|  | T023a | 4 | 1 | - | - | - | - |
|  | T025c | 3 | 1 | - | - | - | - |
|  | T026a | 11 | 2 | 0.891 | 0.005 | 0.964 | 0.009 |
|  | T027b | 10 | 7 | 0.956 | 0.033 | 0.773 | 0.003 |
|  | T029a | 1 | 1 | - | - | - | - |
|  | T033a | 10 | 4 | 0.711 | 0.014 | 0.778 | 0.002 |
|  | T034a | 4 | 1 | - | - | - | - |
|  | T036a | 11 | 2 | 0.818 | 0.016 | 0.618 | 0.008 |
|  | T037b | 4 | 2 | - | - | - | - |
|  | T040c | 10 | 0 | 0.978 | 0.011 | 0.727 | 0.003 |
|  | T043a | 2 | 1 | - | - | - | - |
|  | T061c | 4 | 1 | - | - | - | - |
|  | T064a | 10 | 0 | 0.889 | 0.022 | 0.491 | 0.002 |
|  | T068b | 9 | 1 | - | - | - | - |
| Uganda | U005b | 11 | 0 | 0.982 | 0.027 | 0.864 | 0.011 |
|  | U010b | 1 | 1 | - | - | - | - |
|  | U011b | 2 | 1 | - | - | - | - |
|  | U012a | 10 | 0 | 0.911 | 0.02 | 0.639 | 0.01 |
|  | U013b | 1 | 1 | - | - | - | - |
|  | U020a | 11 | 0 | 0.583 | 0.007 | 0.756 | 0.006 |
|  | U021a | 10 | 0 | 0.378 | 0.002 | 0.844 | 0.015 |
|  | U023a | 11 | 0 | 0.978 | 0.033 | 0.644 | 0.004 |
|  | U024b | 10 | 1 | - | - | - | - |
|  | U025b | 7 | 1 | - | - | - | - |
|  | U027b | 3 | 2 | - | - | - | - |
|  | U028b | 11 | 0 | 1 | 0.025 | 0.2 | 0 |
|  | U030b | 11 | 2 | 0.982 | 0.028 | 0.982 | 0.016 |
|  | U035b | 9 | 1 | - | - | - | - |
|  | U037c | 11 | 1 | 0.978 | 0.019 | 0.836 | 0.005 |
|  | U038b | 3 | 1 | - | - | - | - |
|  | U039a | 4 | 2 | - | - | - | - |
|  | U040b | 4 | 2 | - | - | - | - |
|  | U046b | 11 | 0 | 1 | 0.03 | 0.712 | 0.001 |
|  | U053b | 8 | 2 | - | - | - | - |
|  | Bugoto c | 20 | 1 | 0.884 | 0.008 | 0.774 | 0.004 |
|  | Bukoba c | 20 | 1 | 0.958 | 0.007 | 0.89 | 0.005 |
|  | Lwanika c | 20 | 2 | 0.963 | 0.008 | 0.826 | 0.005 |

Note: sites with *S. mansoni* infection present are highlighted. The (a), (b) and (c) indicate whether the collection site were from either (a) marshlands, (b) the lake edge or from (c) another ecosystem such as a canal, paddy, pond or a hybrid environment.
