# Supplementary material for: Molecular xenomonitoring of Schistosoma mansoni infections in Biomphalaria choanomphala at Lake Victoria, East Africa: Assessing roles of abiotic and biotic factors
Source: PLoS Negl Trop Dis. 2025 Jan 2;19(1):e0012771. doi: 10.1371/journal.pntd.0012771 (PMC11695011; doi:10.1371/journal.pntd.0012771)
Supplement: S4 Table — (DOCX) [file pntd.0012771.s007.docx]

Supplementary table 4. The median (IQR) values of the abiotic factors recorded across the Kenyan (*n=*35), Tanzanian (*n=*82) and Ugandan (*n=*53) sites of Lake Victoria.

|  | Kenya | | Tanzania | | Uganda | |
| --- | --- | --- | --- | --- | --- | --- |
| Temperature (°C) | 28.2 | (27.2 - 29.3) | 26.6 | (25 - 28) | 27.7 | (26.1 - 29) |
| Conductivity (μS) | 191 | (129 - 480) | 144 | (110 - 348) | 107 | (93 - 120) |
| pH | 8.2 | (7.9 - 8.7) | 7.9 | (7.4 - 8.7) | 8.3 | (7.4 - 9.2) |
| Total Dissolved Solids | 105 | (79 - 190) | 90 | (67 - 217) | 63 | (58 - 71) |
| Salinity | 0.4 | (0.4 - 0.4) | 0.4 | (0.4 - 0.4) | 0.4 | (0.3 - 0.4) |
| Fluoride (F^-^) | 0.5 | (0.3 - 0.7) | 0.4 | (0.3 - 0.5) | 0.2 | (0.2 - 0.3) |
| Chloride (Cl^-^) | 9.4 | (6.7 - 25) | 8.5 | (6 - 16) | 9.3 | (5.6 - 13.9) |
| Nitrate (NO_3_^-^) | 0.1 | (0 - 0.4) | 0 | (0 - 0.2) | 0 | (0 - 0.1) |
| Phosphate (PO_4_^3-^) | 0.3 | (0.1 - 0.6) | 0.2 | (0 - 0.7) | 0 | (0 - 0.2) |
| Sulphate (SO_4_^3-^) | 3.1 | (1.7 - 6.6) | 1.3 | (0.6 - 4.2) | 0.5 | (0.4 - 0.9) |
| Sodium (Na^+^) | 16.3 | (12.9 - 30) | 13.3 | (10.4 - 21.3) | 11 | (10.5 - 11.5) |
| Magnesium (Mg_2_^+^) | 2.9 | (2.4 - 6.5) | 2.7 | (2.5 - 5.6) | 2.9 | (2.8 - 3.2) |
| Calcium (Ca_2_^+^) | 8.3 | (6.2 - 22.4) | 10 | (5.6 - 28.7) | 7.2 | (6.8 - 7.8) |
| Potassium (K^+^) | 7.7 | (5.6 - 15.1) | 6.7 | (4.9 -12.5) | 11.1 | (6.4 - 19.6) |
